# Exogenous citric acid, salicylic acid, and putrescine treatments preserve the postharvest quality and physicochemical properties of broccoli (*Brassica oleracea* L. var. *italica*) during cold storage

**DOI:** 10.1002/fsn3.3862

**Published:** 2023-11-27

**Authors:** Hakan Kibar, Beyhan Kibar, Nezahat Turfan

**Affiliations:** ^1^ Department of Seed Science and Technology, Faculty of Agriculture Bolu Abant Izzet Baysal University Bolu Türkiye; ^2^ Department of Horticulture, Faculty of Agriculture Bolu Abant Izzet Baysal University Bolu Türkiye; ^3^ Department of Biology, Faculty of Science Kastamonu University Kastamonu Türkiye

**Keywords:** broccoli sample, color properties, flavonoid, mineral composition, phenolic

## Abstract

To extend the postharvest storage life of broccoli samples (*Brassica oleracea* L. var. *italica*), an exogenous application of citric acid (CA), salicylic acid (SA), and putrescine (PUT) was tested in multiple combinations (0.5 and 1 mM) at 4 ± 0.5°C and 90 ± 5% relative humidity (RH) for 21 days (d). The weight loss (WL), respiration rate (RR), total soluble solids (TSS), pH, color (L*, a*, b*, chroma, and hue angle), proximate and mineral contents, phenolic and flavonoid contents, and other biochemical properties of the treated and untreated broccoli samples were evaluated throughout the storage duration. The lowest WL was observed when exposed to 0.5 mM of PUT. 1 mM CA and PUT treatments were affected by RR, depending on storage conditions. The lowest TSS content was observed in broccoli samples treated with 0.5 mM CA among all treatments. The chroma value of the samples was preserved by the 0.5 mM SA treatment. The most abundant element in broccoli samples was potassium in the control application, followed by a 1 mM SA treatment. In addition, the protein content was the highest in the 1 mM PUT treatment. The highest vitamin C was determined in the 1 mM CA treatment, and the most abundant vanillic acid was found in broccoli exposed to the 0.5 mM and treatment. Glucose content was determined at the lowest level in the 0.5 mM SA treatment, while higher increases occurred in other treatments. In terms of these findings, 1 mM CA, 1 mM SA, and 1 mM PUT delay WL, RR, and color degradation and prolong the storage life of broccoli samples stored at 4 ± 0.5°C. It was concluded that the biochemical content, fresh weight, and green color of broccoli samples throughout postharvest and storage can be maintained longer by exogenous application of these natural compounds. Therefore, we recommend 1 mM PUT and 1 mM CA treatments to maintain the quality of broccoli by minimizing losses in morphological properties, mineral, and biochemical compositions during postharvest storage.

## Graphical abstract

In this study, we aimed to examine the postharvest storage life of broccoli samples (*Brassica oleracea* L. var. *italica*). An exogenous treatment of citric acid (CA), salicylic acid (SA), and putrescine (PUT) was tested in multiple combinations (0.5 and 1 mM) at 4 ± 0.5˚C and 90 ± 5% relative humidity (RH) for 21 days (d). It has been determined that natural compounds such as citric acid (CA), salicylic acid (SA), and putrescine (PUT) are effective on the quality, and reduce quality declines in broccoli during different storage periods.

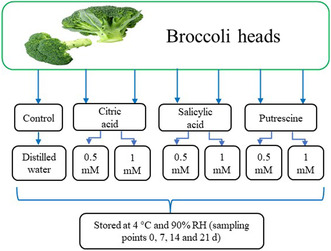

## 1 INTRODUCTION

Broccoli (*Brassica oleracea* L. var. *italica*) is a cool‐climate vegetable belonging to the *Brassicaceae* family and is characterized by green samples and stems (King & Morris, 1994). Vegetables are low‐cost sources of mineral compositions, proteins (Sarker, Oba, et al., 2022; Tarafder et al., 2023), vitamins (Tarafder et al., 2023), phenols, flavonoids (Sarker et al., 2023; Sarker & Ercisli, 2022), colorants with strong antiradical potential (Sarker & Oba, 2021) that contribute to human nutrition (Sarker & Oba, 2021), and health promotion (Sarker, Lin, et al., 2022). Broccoli is a widely enjoyed vegetable globally due to its rich mineral, vitamin, and nutrient content, as highlighted by Singh et al. (2011). Changing lifestyles in recent years have increased the requisition for healthy, useful, additive‐free, safe, and nutritious foods. The fresh‐cut industry has replied to these requisitions (James et al., 2010). There are many factors that affect the quality and shelf life of freshly cut vegetables. These factors are determined by many pre‐harvest conditions, from cultural practices to fertilization and irrigation (Colelli & Elia, 2009) and postharvest storage conditions (Tarafder et al., 2023).

In recent years, natural compounds such as polyamines (putrescine, spermine, and spermidine), salicylic acid, and citric acid have been used, in addition to different preservation techniques, to decrease postharvest quality losses and extend the storage life of vegetables. Studies investigating the effects of these compounds on the storage time and quality of different vegetables postharvest have gained intensity. By treating vegetables with some low‐dose chemical solutions postharvest, quality can be maintained and an increase in storage time can be achieved. However, the activities of these compounds vary depending on many factors, such as species, variety, environmental conditions, plant development period, application method, duration, and concentration (Alali et al., 2023; Rastegar et al., 2022; Razzaq et al., 2013). Putrescine (PUT), one of the substances used to maintain or improve postharvest quality in products, is a low‐molecular‐weight organic compound found in almost all living organisms (Khosroshahi et al., 2007). Şahin (2022) and Alali et al. (2023) reported that exogenous PUT treatments have positive effects on fruit (for example, retardation of ripening and aging, slowing of fruit softening, inhibiting ethylene production and activity, and controlling the amount of water‐soluble dry matter and titratable acid content). Salicylic acid (SA) serves as an intrinsic plant growth regulator, playing a pivotal role in governing various physiological processes in plants, thereby influencing their growth and development (Hayat et al., 2010). SA, being a naturally occurring and safe phenolic compound, holds promise in mitigating postharvest losses in horticultural crops. It has the capability to prolong the shelf life of fruits and vegetables after harvesting, all the while preserving their sensory and nutritional attributes (Asghari & Aghdam, 2010). It has been determined that SA applied exogenously to horticultural crops is effective in maintaining postharvest quality (Asghari & Aghdam, 2010; Davarynejad et al., 2015; Dokhanieh & Aghdam, 2016; Peng & Jiang, 2006). Citric acid (CA) is an organic acid, and it increases the postharvest storage life and affects the physiological and biochemical quality of horticultural products. CA is widely used as a preservative in the food industry. It is one of the chemicals used to prevent darkening (Garcia & Barrett, 2002). Pizzocaro et al. (1993) noted that the enzyme polyphenol oxidase (PPO), which plays a role in enzymatic darkening, is inhibited by CA.

In the literature, various studies have shown that polyamine, salicylic acid, and citric acid treatments applied exogenously to fruit and vegetables postharvest indirectly delay the losses in quality parameters in fruit and vegetables (Hafiz et al., 2017; Mustafa et al., 2018a; Mustafa et al., 2018b; Surendran et al., 2018). However, there are limited studies in the literature on the application of these treatments to broccoli. Therefore, the aim of this work was to investigate the effects of exogenous CA, SA, and PUT treatments on weight loss, respiration rate, soluble solids content, pH, color, proximate and mineral contents, phenolic and flavonoid contents, and other biochemical quality properties of samples of the broccoli cultivar stored at 4 ± 0.5°C and 90 ± 5% relative humidity for 21 days.

## 2 MATERIALS AND METHODS

### 2.1 Sample preparation, treatments, and experimental design

Sixty kg of broccoli samples (*Brassica oleracea* L. var. *italica*), cultivar Marathon F_1_, were purchased from a producer in Bolu city (Türkiye). Broccoli samples were harvested at commercial maturity (before flower buds opened) and carefully transferred to the laboratory within 4 h. The experiment was conducted in a completely randomized design, including seven treatments (Control; 0.5 and 1.0 mM CA; 0.5 and 1.0 mM SA; and 0.5 and 1.0 mM PUT; Table 1). We chose these three applications and their treatment doses by taking into account the studies on vegetables in the literature. The utilization of various natural compounds, including CA, SA, and PUT treatments, aims to elucidate the impact of eventual storage degradation on the physicochemical and quality attributes of broccoli. This research was structured following a trial plan involving three replicates, with each replicate comprising a 700 g sample of broccoli. Broccoli samples were prepared by separating flowers and stems. The samples were immersed in CA (0.5 and 1.0 mM), SA (0.5 and 1.0 mM), and PUT (0.5 and 1.0 mM) solutions (2 L, 20°C) as well as distilled water (control), for 5 min, and then dried on blotting papers for 30 min. Broccoli samples, including those treated with CA, SA, and PUT, as well as untreated (control) samples, were moved into 2 L plastic containers weighing 700 g each. These containers were then positioned on shelves within a cold storage environment maintained at 4 ± 0.5°C and 90 ± 5% relative humidity. Measurements were conducted on days 7, 14, and 21 from the start of the storage period.

**TABLE 1:** Treatments used in the study, their contents, and abbreviations.

Treatment	Content	Abbreviation
Control	Control	Control
1	0.5 mM Citric acid	0.5 mM CA
2	1.0 mM Citric acid	1.0 mM CA
3	0.5 mM Salicylic acid	0.5 mM SA
4	1.0 mM Salicylic acid	1.0 mM SA
5	0.5 mM Putrescine	0.5 mM PUT
6	1.0 mM Putrescine	1.0 mM PUT

*Note*: Uppercase letters indicate differences in storage duration and treatments. Lowercase letters indicates difference in SD × T interaction.

### 2.2 Weight loss, respiration rate, soluble solids content, and pH

The percentage of weight loss (WL) in broccoli samples was determined with precision using a sensitive balance (ISOLAB‐602.01.033, İstanbul, Türkiye) with a precision of 0.01 g. WL in the broccoli samples was then computed according to Equation 1, as outlined in the study by Boonsiriwit et al. (2020).

(1) $$\mathrm{WL}=\frac{\left(W_{i}-W_{f}\right)}{W_{i}}\times 100$$

where;

*W*_*i*_ = weight of broccoli samples before storage, g

*W*_*f*_ = weight of broccoli samples at 7, 14 and 21 days of storage, g

In order to assess respiration rate (RR), we introduced 150 g of broccoli from each replication into 2 L glass containers. A 2 cm‐diameter aperture was created in the plastic lid of the glass container. The CO_2_ measuring device probe (Testo 535 CO2 Meter, Germany) was then inserted through this opening. Then, the area where the probe contacted the lid was tightly covered with parafilm to prevent air from entering the glass bottle and CO_2_ from escaping from the bottle. All measurements were made in a climate room with a controlled temperature (4 ± 0.5°C) and relative humidity (90 ± 5%). RR was calculated according to Equations (2–4) and expressed as mg CO_2_ kg^−1^ h^−1^.

(2) $$\mathrm{RR}=\frac{{∆}_{{\mathrm{CO}}_{2}}\times M_{{\mathrm{CO}}_{2}}\times V_{h}}{V_{m}\times m\times ∆t}$$

(3) $${∆}_{{\mathrm{CO}}_{2}}={\mathrm{CO}}_{2\left(t2\right)}-{\mathrm{CO}}_{2\left(t1\right)}$$

(4) $$V_{m}=\frac{R\times T}{P}$$

where;

${∆}_{{\mathrm{CO}}_{2}}$: CO_2_ volumetric concentration, mg kg^−1^, 10^−6^ L L^−1^

$M_{{\mathrm{CO}}_{2}}$: Molecular weight of CO_2_ gas, 44.01 g mol^−1^

$V_{h}$: Flask volume, L

m: The weight of samples, kg

∆t: Duration of the experiment, h

${\mathrm{CO}}_{2\left(t1\right)}$: CO_2_ concentration in the initial, mg kg^−1^, 10^−6^ L L^−1^

${\mathrm{CO}}_{2\left(t2\right)}$: CO_2_ concentration at the end of the experiment, mg kg^−1^, 10^−6^ L L^−1^

$V_{m}$: Molar volume of gas, L mol^−1^

R: Gas constant, 0.08206 L^−1^ mol^−1^ K^−1^

T: Temperature, K

P: Pressure, atm

To determine the total soluble solids content (TSS), 10 g of a broccoli sample consisting of flowers and stems was blended using a blender and the water was extracted. TSS was determined using an ATC BX50 (Turkey) refractometer at 23 ± 1.5°C and expressed as %.

For pH measurement, broccoli samples were kept in 250 mL of pure water in a beaker at room temperature (23 ± 1.5°C) for 24 h. Then, the pH leakage of the samples was measured with the help of a pH meter (Thermo, OrionStar A111, USA).

### 2.3 Color measurements

The color data (L*, a*, and b*) of samples were obtained via a digital colorimeter (3NH NR60CP, Shenzhen, China) in five replicates during storage (Kibar & Kibar, 2017). The chroma and hue angle parameters were obtained using Equations (5) and (6), respectively.

(5) $$\text{Chroma}=\sqrt{a^{2}+b^{2}}$$

(6) $$\mathrm{Hue}\;\text{angle}=\arctan\left(\frac{b}{a}\right)$$

### 2.4 Proximate and mineral composition

The ash content was obtained by burning the dried samples in an ash oven (Mipro MKF, Ankara, Türkiye) at 550°C for about 8 h until gray‐white ash was obtained (AOAC, 1990).

All the samples underwent drying in an oven at 65°C until reaching a consistent weight. Subsequently, 10 g of the dried samples were ground into a powder using a laboratory mill. The protein content was determined following the Kjeldahl method (Sarker & Oba, 2019) to determine nitrogen. The nitrogwn content of broccoli samples underwent conversion to ammonium sulfate using sulfuric acid and a catalyst mixture through digestion at 380°C. The released ammonia was distilled alongside a sodium hydroxide solution for absorption by boric acid, followed by titration and the calculation of the nitrogen percentage using the specified formula. The total nitrogen (N) was determined using the Kjeldahl method, with a conversion factor of 6.25 applied for the broccoli samples, as specified in AOAC (1990).

(7) $$\text{Protein content}\;\left(\%\right)=\left[\left\{\left(A_{1}-A_{2}\right)\times N\times 14.01\times 100\right\}/\left(W\times 1000\right)\right]\times 6.25$$

where;

A_1_: The quantity of sulfuric acid employed in neutralizing the sample

A_2_: The amount of acid utilized in neutralizing the blank

N: The normality of the titrant, which is a standard hydrochloric acid with a concentration of 0.1 N.

W: Broccoli sample weight, g

The value of 14.01 in the equation is the molecular weight of nitrogen.

We determined phosphorus (P), potassium (K), magnesium (Mg), and calcium (Ca) from powdered samples following the nitric‐perchloric acid digestion method (Sarker, Oba, et al., 2022). For this digestion, 40 mL of HClO_4_ (70%), 10 mL of H_2_SO_4_ (96%), and 400 mL of HNO_3_ (65%) were added to the 0.5 g dried broccoli sample in the presence of carborundum beads. We read the absorbance by using inductively coupled plasma‐optical emission spectrometry (ICP‐OES; Thermo Scientific, X Series, Cambridge, UK) at wavelengths of 178.2 nm P, 766.5 nm K, 285.2 nm Mg, and 396.9 nm Ca content. Mineral and protein contents were reported as milligrams per 100 grams of dry weight (DW) [mg 100 g^−1^ DW] and as percentages (%), respectively.

### 2.5 Biochemical measurements

Broccoli sample extracts (100 g) were enriched with 2.5% (w v^−1^) metaphosphoric acid (Sigma, M6285, 33.5%, Taufkirchen, Germany), then subjected to centrifugation at 2362*g* for 10 min at 4°C. Subsequently, 0.5 mL of the resulting mixture was adjusted to a final volume of 10 mL with 2.5% (w v^−1^) metaphosphoric acid. The supernatants were subsequently filtered through a 0.45 μm PTFE syringe filter (Millex‐HV Hydrophilic PVDF, Millipore, Taufkirchen, Germany). For the identification of ascorbic acid at 25°C, a C18 column (Phenomenex Luna C18, 250 × 4.60 mm, 5 μm) was employed. The mobile phase used was double‐distilled water with a flow rate of 1 mL min^−1^, adjusted to pH 2.2 (acidified with H_2_SO_4_). Spectral measurements were conducted at a wavelength of 254 nm using a DAD detector. Various L‐ascorbic acid standards (Sigma A5960) at concentrations of 50, 100, 500, 1000, and 2000 ppm were utilized to quantify the readings for ascorbic acid (Cemeroglu, 2007). The results were reported as mg per 100 g of fresh weight (FW) [mg 100 g^−1^ FW].

The modified HPLC procedure (Rodriguez‐Delgado et al., 2001) was employed to detect phenolic acids such as ferulic acid, gallic acid, vanillic acid, caffeic acid, and chlorogenic acid, as well as the flavonoid quercetin in broccoli samples. Phenolic compound standards for ferulic, vanillic, and caffeic acids were prepared at concentrations of 50, 100, 150, 200, 250, and 300 ppm, while the gallic acid standard was prepared at concentrations of 20, 40, 60, 80, 100, and 120 ppm. The chlorogenic acid standard was prepared at concentrations of 200, 400, 600, 800, 1000, and 1200 ppm. All standards were sourced from Sigma‐Aldrich with a purity of 99% from Taufkirchen, Germany. Additionally, the flavonoid content standard, quercetin, was prepared at concentrations of 100, 200, 300, 400, 500, and 600 ppm from the same source. For the analysis, 15 g of samples were initially chosen from each replicate and homogenized using a food‐grade blender. Subsequently, the homogenized samples were centrifuged at 8050*g* at 4°C for 30 min. The resulting supernatants were then filtered first through coarse filter paper and then through a 0.45 μm membrane filter (Millipore Millex‐HV Hydrophilic PVDF; Millipore, Massachusetts, USA) before being injected into an HPLC system from Agilent in California, USA. Chromatographic separation was accomplished using a 250 × 4.6 mm, 4 μm ODS (Octadecyl‐silica) column from HiChrom in Leicestershire, UK. The mobile phase for separating phenolics and flavonoids from the broccoli samples consisted of Solvent A (methanol:acetic acid:water, 10:2:28) and Solvent B (methanol:acetic acid:water, 90:2:8). UV spectral measurements were recorded at 254 and 280 nm, and the flow rate and injection volume were set at 1 mL min^−1^ and 20 μL, respectively (Taş et al., 2022).

The determination of anthocyanin content followed the procedure outlined by Mancinelli (1990). Initially, a 500 mg sample was extracted using 3 mL of methanol‐HCl solution (1% HCl, v v^−1^), and the resulting homogenates were refrigerated at 3–5°C for 48 h with periodic agitation. Subsequently, the extracts were filtered, and the anthocyanin content was quantified at a wavelength of 530 nm. Anthocyanin concentration within the samples was computed using the provided formula, utilizing an extinction coefficient of 31.6 M^−1^ cm^−1^.

(8) $$\text{Anthocyanin}\;\left(\text{μmol}\;g^{-1}\right)=\left[\left(A530–0.33\times A657\right)/31.6\right]\times \left(V/m\right)$$

V: volume of solution used, mL

m: amount of sample, g

The method of Spies (1957) was employed to determine the total free amino acid content in broccoli samples. Initially, 21 g of citric acid monohydrate was dissolved in 200 mL of a 1 M NaOH solution (adjusted to pH 5.5). Distilled water was then added to bring the final volume to 500 mL. Subsequently, 125 mL of this prepared solution was extracted, and 0.2 g of SnCl_2_‐2H_2_O was introduced into it. In a separate step, 5 g of ninhydrin was dissolved in 125 mL of ethanol, and this ninhydrin solution was combined with the previous solution, forming reagent solution No. 1. The samples were subjected to drying in an oven at 60°C until a constant weight was achieved. Next, a 1 g dry powder sample was homogenized with 25 mL of distilled water for 30 min using a shaker. Following homogenization, determinations were performed using 1 mL of the resulting extract, which was filtered first through coarse filter paper and then through blue‐band filter paper. To this filtered extract, 2 mL of reagent solution No. 1 was added. The mixture was then heated in a water bath at 90–100°C for 10–15 min and subsequently allowed to cool. After cooling, 3 mL of propanol diluted with water at a 1:1 ratio (v v^−1^) was introduced into the cooled tubes. The absorbance of the resulting blue‐violet‐colored solution was measured using a spectrophotometer (Cecil Elegant Technology CE 5502 Scanning double beam UV SP. 5000 Series; Cecil Instruments Ltd., Cambridge, UK) at 570 nm. To create standards, glycine was used in concentrations ranging from 0.05 to 0.5 mM, and the same processing steps were applied to these standards. The free amino acid content was reported as mg 100 g^−1^ of dry weight (DW).

Proline content in broccoli samples was determined following the procedure outlined by Bates et al. (1973). Initially, a 0.5 g dried powdered sample was crushed in a mortar using 5 mL of a 3% sulfosalicylic acid solution. Subsequently, the mixture was filtered into 15 ‐mL tubes. The mortar was then rinsed with an additional 5 mL of the 3% sulfosalicylic acid solution, which was also filtered into a tube. The final volume was adjusted to 10 mL, followed by brief vortexing. The resulting mixture was further filtered through cheesecloth, and 2 mL of the filtrate was transferred to 25 mL glass tubes. To this, 2 mL of ninhydrin and 2 mL of glacial acetic acid (acidic ninhydrin) were added. The tubes were sealed with parafilm and punctured several times with a pin. These prepared samples were boiled at 90–100°C for 1 h and then cooled in an ice bath. Toluene, pre‐cooled in the refrigerator overnight or cooled in an ice bath for 1 h, was introduced into the cooled samples, resulting in the formation of two separate phases. The upper phase of the toluene‐added samples was transferred to quartz tubes, and the absorbance was measured at 520 nm. Toluene served to stabilize the spectrophotometer. Proline standards were also prepared, and the proline content was calculated using the derived equation (*X* = *Y* − 0.092/0.1584). Proline content was reported mg 100 g^−1^ of dry weight (DW).

The Anthrone method was employed to measure the total carbohydrate, glucose, and sucrose levels. Initially, 1 g of the powdered sample was introduced into a 50 mL flask, followed by the addition of 50‐mL of 80% ethanol. This mixture was left to incubate at 4°C for 24 h. Subsequently, the suspensions underwent filtration through Whatman No. 4 filter paper. The first filtrate was collected and subjected to a 60°C water bath to evaporate the ethanol under reduced pressure; this filtrate was then utilized for glucose determination. To assess sucrose levels, the remaining sample residue was incubated in 30 mL of 52% perchloric acid at 4°C for 24 h. Afterward, the resulting suspension was filtered using Whatman No. 4 filter paper, and the resulting filtrate was used for both sucrose and total carbohydrate content determination. The quantification of glucose and sucrose was accomplished by referencing standard calibration curves for glucose and sucrose (Pearson et al., 1976). The results for total carbohydrate, glucose, and sucrose content were expressed as mg 100 g^−1^ of dry weight (DW).

### 2.6 Statistical analysis

The experimental design was performed according to a complete randomized design (CRD) with three replicates (*n* = 3). Experimental data were given as the mean ± standard deviation. Data were performed for a two‐factor analysis of variance to define the effects of the main factors and interactions (SD × T), wherein storage duration (SD) and treatments (T) were regarded as the main factors (JMP 13.2; SAS Institute Inc., Cary, North Carolina, USA). The comparisons among mean values were performed using Tukey's HSD test (*p* < .05). The correlation matrix and hierarchical clustering heat map dendogram were used to clarify the relationship among of treatments with examined properties in this study using the metan and heatmap functions in R software (version 4.2.3).

## 3 RESULTS

### 3.1 Changes in weight loss, respiratory rate, soluble solids content, and pH content

Weight loss (WL) was influenced by the *SD*, *T*, and *SD* × *T* interactions, as shown in Table 2. A remarkable increase was observed in the control group on day 21. The increase in WL was higher with longer storage times. When the treatments were examined, the highest WL was found in the control group, while the lowest was determined in the 0.5 mM PUT and 1 mM PUT treatments.

**TABLE 2:** Effect of citric acid, salicylic acid, and putrescine on WL, RR, TSS, and pH contents of broccoli sample during storage at 4°C.

Storage duration (SD)	WL (%)	RR (mg CO_2_ kg^−1^ h^−1^)	TSS (%)	pH
Before storage	0.00 ± 0.00 D	91.6 ± 9.07 AB	9.00 ± 0.38 D	6.40 ± 0.08 C
Day 7	0.48 ± 0.12 C	96.3 ± 7.48 B	10.0 ± 0.41 C	7.14 ± 0.09 A
Day14	0.95 ± 0.24 B	100 ± 8.84 AB	10.8 ± 0.46 B	6.39 ± 0.08 C
Day 21	1.50 ± 0.47 A	104 ± 10.1 A	11.6 ± 0.56 A	6.77 ± 0.16 B
*Treatment* (*T*)				
Before storage	0.00 ± 0.00 B	91.6 ± 9.07 B	9.00 ± 0.38 B	6.40 ± 0.08 ns
Control	1.32 ± 0.84 A	119 ± 7.71 A	11.5 ± 1.10 A	6.75 ± 0.33 ns
0.5 mM CA	1.29 ± 0.58 A	100 ± 5.61 B	10.3 ± 0.80 AB	6.80 ± 0.39 ns
1 mM CA	0.90 ± 0.46 AB	95.2 ± 3.52 B	10.6 ± 0.90 A	6.78 ± 0.38 ns
0.5 mM SA	0.80 ± 0.31 AB	99.7 ± 5.72 B	10.6 ± 0.64 A	6.76 ± 0.35 ns
1 mM SA	0.95 ± 0.42 AB	96.5 ± 3.64 B	10.8 ± 0.71 A	6.83 ± 0.33 ns
0.5 mM PUT	0.79 ± 0.35 AB	96.3 ± 4.03 B	10.7 ± 0.70 A	6.70 ± 0.32 ns
1 mM PUT	0.79 ± 0.29 AB	95.2 ± 3.19 B	11.1 ± 0.60 A	6.75 ± 0.33 ns
*SD* × *T*				
Before storage	0.00 ± 0.00 h	91.7 ± 9.07 f	9.00 ± 0.38 h	6.40 ± 0.08 f‐i
Day 7 × Control	0.45 ± 0.02 gh	112 ± 6.50 bc	10.3 ± 0.41 efg	7.14 ± 0.06 abc
Day 14 × Control	1.15 ± 0.13 cde	120 ± 4.04 ab	11.4 ± 0.36 bcd	6.39 ± 0.05 ghi
Day 21 × Control	2.35 ± 0.28 a	126 ± 4.93 a	12.8 ± 0.32 a	6.74 ± 0.07 d–h
Day 7 × 0.5 mM CA	0.68 ± 0.21 efg	96.3 ± 4.04 def	9.57 ± 0.35 gh	7.16 ± 0.06 abc
Day 14 × 0.5 mM CA	1.32 ± 0.33 cd	99.0 ± 2.64 def	10.2 ± 0.36 fg	6.45 ± 0.06 e–i
Day 21 × 0.5 mM CA	1.88 ± 0.34 ab	106 ± 3.68 cd	11.3 ± 0.38 b–e	6.80 ± 0.46 cde
Day 7 × 1 mM CA	0.44 ± 0.15 gh	92.0 ± 2.64 f	9.57 ± 0.21 gh	7.20 ± 0.09 ab
Day 14 × 1 mM CA	0.81 ± 0.17 d–g	95.6 ± 2.62 def	10.8 ± 0.21 b–f	6.35 ± 0.09 hi
Day 21 × 1 mM CA	1.47 ± 0.02 bc	98.7 ± 2.80 def	11.5 ± 0.38 bc	6.79 ± 0.09 c–f
Day 7 × 0.5 mM SA	0.47 ± 0.09 fgh	94.6 ± 2.82 def	10.0 ± 0.36 fg	7.16 ± 0.09 abc
Day 14 × 0.5 mM SA	0.80 ± 0.13 efg	97.6 ± 3.21 def	10.7 ± 0.30 c–f	6.37 ± 0.06 ghi
Day 21 × 0.5 mM SA	1.13 ± 0.18 cde	105 ± 4.06 cde	11.3 ± 0.31 bcd	6.75 ± 0.10 d–g
Day 7 × 1 mM SA	0.47 ± 0.04 gh	93.8 ± 3.15 def	10.1 ± 0.26 fg	7.22 ± 0.09 a
Day 14 × 1 mM SA	0.98 ± 0.13 c–f	96.8 ± 3.15 def	10.8 ± 0.30 b–f	6.47 ± 0.08 e–i
Day 21 × 1 mM SA	1.40 ± 0.11 bc	99.0 ± 3.61 def	11.6 ± 0.25 bc	6.82 ± 0.08 b–e
Day 7 × 0.5 mM PUT	0.41 ± 0.04 gh	92.7 ± 1.77 f	9.97 ± 0.15 fgh	7.04 ± 0.09 a–d
Day 14 × 0.5 mM PUT	0.81 ± 0.15 d–g	96.9 ± 2.00 def	10.7 ± 0.36 c–f	6.32 ± 0.07 i
Day 21 × 0.5 mM PUT	1.16 ± 0.21 cde	100 ± 2.87 c–f	11.5 ± 0.35 bcd	6.74 ± 0.07 d–h
Day 7 × 1 mM PUT	0.47 ± 0.01 gh	92.2 ± 1.96 f	10.5 ± 0.17 d–g	7.10 ± 0.07 a–d
Day 14 × 1 mM PUT	0.79 ± 0.04 efg	95.2 ± 2.61 def	11.3 ± 0.31 b–e	6.36 ± 0.12 ghi
Day 21 × 1 mM PUT	1.20 ± 0.10 cde	97.5 ± 2.98 def	11.7 ± 0.32 b	6.80 ± 0.10 cde
*Significant effects* (*p values*)				
SD	0.002	0.008	0.001	0.001
T	0.004	0.001	0.001	0.767
SD × T	0.001	0.001	0.001	0.001

*Note*: Uppercase letters indicate differences in storage duration and treatments. Lowercase letters indicates difference in SD × T interaction.

Abbreviations: ±, standard deviation of mean; CA, citric acid; PUT, putrescine; RR, respiratory rate; SA, salicylic acid; TSS, total soluble solids; WL, weight loss.

Depending on the increase in SD, RR showed an increasing trend compared to pre‐storage. The highest increase occurred on 21 days. In all postharvest treatments, the respiratory rate was found to be lower than the control, and statistical changes were not observed among treatments. When the SD × T interaction was examined, the highest respiratory rates occurred in the control group (Table 2).

Irrespective of CA, SA, and PUT treatments, the TSS of the broccoli samples increased during the storage period. At the end of the 21‐d storage period of broccoli samples, the TSS content showed the highest increase with 22.4% compared to pre‐storage. In terms of treatments, when TSS values were compared with before storage, the highest value was found in the control group, and the lowest value was detected in the 0.5 mM CA treatment. In terms of SD × T interaction, TSS values increased with increasing storage duration in each treatment. Generally, the highest values in each treatment were determined on the 21st d of storage. While the highest TSS value was found on Day 21 × Control, the lowest value was observed before storage (Table 2).

In terms of SD, pH contents fluctuated compared to pre‐storage. There was no statistical difference since there was not much variation among treatments. SD × T interaction was found to be statistically significant, and fluctuations occurred in pH contents. Compared with pre‐storage, the highest increase was found in Day 7 × 1 mM SA with 12.8%, and the decrease was found in Day 14 × 0.5 mM PUT with 1.27% (Table 2).

### 3.2 Changes in color properties

Differences among the color properties examined in terms of SD and SD × T interactions were found to be statistically significant. When SD increased, L*, b*, and chroma increased while a* values decreased. The hue angle fluctuated with increasing SD. In terms of SD × T interaction, the increases in color values except for the a* value were generally observed with increasing SD for each treatment. However, in some treatments, fluctuations have emerged instead of an increase. The statistical difference among treatments in terms of all color properties examined was found to be insignificant. Although there were no statistical differences, the highest values were determined in the control group (Table 3).

**TABLE 3:** Effect of citric acid, salicylic acid, and putrescine on color properties of broccoli sample during storage at 4°C.

Storage duration (SD)	L*	a*	b*	Chroma	Hue angle
Before storage	42.6 ± 5.71 C	−3.47 ± 1.22 AB	17.3 ± 6.02 BC	17.7 ± 6.68 BC	99.5 ± 6.62 ns
Day 7	42.9 ± 4.34 C	−4.67 ± 2.51 A	19.5 ± 4.44 C	20.1 ± 4.82 C	102 ± 4.38 ns
Day14	47.9 ± 3.64 B	−5.36 ± 2.83 AB	22.5 ± 4.75 AB	23.2 ± 5.05 AB	103 ± 5.40 ns
Day 21	50.4 ± 2.55 A	−6.58 ± 2.72 B	24.1 ± 3.09 A	25.1 ± 3.46 A	101 ± 16.7 ns
*Treatment* (*T*)					
Before storage	42.67 ± 5.71 ns	−3.47 ± 1.22 ns	17.3 ± 6.02 ns	17.7 ± 6.68 ns	99.5 ± 6.62 ns
Control	48.7 ± 3.28 ns	−6.83 ± 2.88 ns	23.6 ± 3.34 ns	24.7 ± 3.60 ns	105 ± 5.43 ns
0.5 mM CA	46.1 ± 4.87 ns	−5.36 ± 2.53 ns	21.2 ± 4.25 ns	22.0 ± 4.67 ns	103 ± 4.29 ns
1 mM CA	47.0 ± 5.11 ns	−4.31 ± 3.20 ns	22.1 ± 5.11 ns	22.6 ± 5.53 ns	98.5 ± 9.67 ns
0.5 mM SA	47.8 ± 5.94 ns	−5.51 ± 1.94 ns	22.9 ± 4.98 ns	23.6 ± 5.17 ns	103 ± 3.64 ns
1 mM SA	45.8 ± 5.65 ns	−5.52 ± 3.18 ns	20.8 ± 5.50 ns	22.3 ± 3.42 ns	103 ± 5.36 ns
0.5 mM PUT	46.8 ± 3.99 ns	−5.78 ± 3.37 ns	22.1 ± 5.07 ns	23.6 ± 5.17 ns	97.8 ± 11.2 ns
1 mM PUT	46.9 ± 4.06 ns	−5.45 ± 1.96 ns	21.5 ± 3.23 ns	21.6 ± 6.03 ns	103 ± 4.18 ns
*SD* × *T*					
Before storage	42.6 ± 5.71 b‐e	−3.47 ± 1.22 d	17.3 ± 6.02 ab	17.7 ± 6.68 c	99.5 ± 6.62 ab
Day 7 × Control	46.5 ± 3.14 a‐e	−7.00 ± 2.46 ab	22.5 ± 3.15 ab	23.6 ± 3.69 ab	104 ± 3.00 a
Day 14 × Control	47.6 ± 2.31 a‐e	−5.17 ± 3.29 c	23.0 ± 4.47 ab	23.7 ± 4.51 ab	103 ± 7.29 a
Day 21 × Control	51.9 ± 1.37 a	−8.31 ± 2.43 a	25.4 ± 1.73 ab	26.8 ± 1.82 a	108 ± 4.95 ab
Day 7 × 0.5 mM CA	42.4 ± 6.43 cde	−3.81 ± 2.68 d	19.7 ± 6.13 ab	20.1 ± 6.55 b	100 ± 3.55 ab
Day 14 × 0.5 mM CA	47.1 ± 3.27 a‐e	−5.78 ± 2.63 c	21.3 ± 4.14 ab	22.2 ± 4.67 ab	104 ± 3.89 a
Day 21 × 0.5 mM CA	49.0 ± 1.43 a‐d	−6.49 ± 1.81 b	22.7 ± 1.67 ab	23.6 ± 1.91 ab	105 ± 3.76 ab
Day 7 × 1 mM CA	41.2 ± 2.81 de	−2.93 ± 1.35 e	18.7 ± 3.06 ab	18.9 ± 3.02 c	99.1 ± 4.25 ab
Day 14 × 1 mM CA	49.6 ± 3.98 abc	−4.02 ± 3.73 d	23.8 ± 7.11 ab	24.2 ± 7.56 a	98.6 ± 6.03 ab
Day 21 × 1 mM CA	50.2 ± 2.01 abc	−5.99 ± 3.73 b	23.8 ± 3.04 ab	24.7 ± 3.90 a	97.9 ± 16.5 a
Day 7 × 0.5 mM SA	41.2 ± 3.99 de	−4.48 ± 2.21 cd	17.0 ± 3.44 b	17.6 ± 3.84 c	103 ± 5.18 a
Day 14 × 0.5 mM SA	50.1 ± 3.48 abc	−5.09 ± 1.77 c	25.1 ± 1.34 ab	25.6 ± 1.64 a	101 ± 3.27 ab
Day 21 × 0.5 mM SA	52.3 ± 3.05 a	−6.96 ± 0.98 b	26.7 ± 2.22 a	27.6 ± 2.36 a	104 ± 1.23 a
Day 7 × 1 mM SA	40.0 ± 4.02 e	−3.22 ± 1.57 d	17.0 ± 5.69 ab	17.4 ± 5.84 c	100 ± 3.03 ab
Day 14 × 1 mM SA	46.1 ± 3.15 a‐e	−4.47 ± 2.52 cd	19.2 ± 3.49 ab	19.8 ± 3.82 b	102 ± 5.73 ab
Day 21 × 1 mM SA	51.3 ± 2.48 a	−8.87 ± 2.13 a	26.2 ± 1.99 ab	27.7 ± 2.32 a	108 ± 3.71 a
Day 7 × 0.5 mM PUT	44.0 ± 3.24 a‐e	−5.94 ± 3.19 b	21.5 ± 4.75 ab	22.4 ± 5.37 ab	104 ± 5.24 a
Day 14 × 0.5 mM PUT	49.3 ± 5.01 a‐d	−8.13 ± 3.01 a	23.9 ± 7.00 ab	25.3 ± 7.53 a	108 ± 2.83 a
Day 21 × 0.5 mM PUT	47.1 ± 1.59 a‐e	−3.27 ± 2.39	20.9 ± 3.49 ab	21.3 ± 3.65 b	80.7 ± 36.1 b
Day 7 × 1 mM PUT	44.7 ± 4.29 a‐e	−5.30 ± 2.10 c	20.4 ± 3.18 ab	21.1 ± 3.54 b	104 ± 3.79 a
Day 14 × 1 mM PUT	45.2 ± 2.72 a‐e	−4.90 ± 2.07 cd	21.3 ± 3.24 ab	21.9 ± 3.41 b	102 ± 4.92 ab
Day 21 × 1 mM PUT	50.7 ± 2.11 ab	−6.14 ± 1.92 b	22.9 ± 3.44 ab	23.8 ± 3.47 ab	105 ± 4.34 a
*Significant effects* (*p values*)					
SD	0.001	0.012	0.001	0.001	0.867
T	0.371	0.285	0.266	0.274	0.385
SD × T	0.001	0.005	0.002	0.002	0.032

*Note*: Uppercase letters indicate differences in storage duration and treatments. Lowercase letters indicates difference in SD × T interaction.

Abbreviations: ±, standard deviation of mean; CA, citric acid; PUT, putrescine; SA, salicylic acid.

### 3.3 Changes in proximate and mineral composition

The initial ash content of samples was found to be 8.19%, and it increased linearly with increasing SD. When compared to before storage, broccoli samples with different treatments showed a significant change in ash content. However, the different treatments and the control group in terms of ash content were statistically in the same group. When the SD × T interaction was examined, ash contents fluctuated and significant differences were observed. The highest ash content was observed in Day 21 × Control and Day 21 × 0.5 mM CA (10.9%) treatments, while the lowest value was dtermined before storage (8.19%) (Table 4).

**TABLE 4:** Effect of citric acid, salicylic acid, and putrescine on proximate and mineral composition of broccoli sample during storage at 4°C.

Storage duration (SD)	Ash (%)	Protein (%)	P (mg 100 g^−1^)	K (mg 100 g^−1^)	Mg (mg 100 g^−1^)	Ca (mg 100 g^−1^)
Before storage	8.19 ± 0.15 C	25.7 ± 0.55 ns	852 ± 3.79 AB	3651 ± 65.5 ns	193 ± 9.71 ns	631 ± 49.1 ns
Day 7	9.69 ± 0.62 B	25.2 ± 2.03 ns	965 ± 83.2 A	3508 ± 162 ns	186 ± 10.8 ns	781 ± 101 ns
Day14	9.76 ± 0.21 AB	25.9 ± 1.70 ns	876 ± 111 AB	3481 ± 317 ns	184 ± 9.06 ns	726 ± 178 ns
Day 21	10.15 ± 0.57 A	25.8 ± 2.15 ns	835 ± 150 B	3455 ± 496 ns	182 ± 11.8 ns	737 ± 231 ns
*Treatment* (*T*)						
Before storage	8.19 ± 0.15 B	25.7 ± 0.55 AB	852 ± 3.79 ABC	3651 ± 65.5 ABC	193 ± 9.71 ns	631 ± 49.1 AB
Control	9.97 ± 0.76 A	24.5 ± 2.68 B	772 ± 186 C	3891 ± 163 A	175 ± 13.8 ns	645 ± 64.5 B
0.5 mM CA	10.2 ± 0.62 A	25.8 ± 0.68 AB	920 ± 98.1 ABC	3260 ± 215 CD	188 ± 8.64 ns	704 ± 77.8 B
1 mM CA	9.69 ± 0.35 A	26.3 ± 1.01 AB	1037 ± 74.1 A	3435 ± 64.3 BC	188 ± 8.00 ns	922 ± 140 A
0.5 mM SA	10.0 ± 0.28 A	24.8 ± 1.63 AB	837 ± 100 BC	3022 ± 417 D	181 ± 9.44 ns	925 ± 206 A
1 mM SA	9.63 ± 0.53 A	25.9 ± 0.64 AB	964 ± 85.4 AB	3771 ± 75.4 A	187 ± 11.1 ns	695 ± 69.3 B
0.5 mM PUT	9.59 ± 0.17 A	24.9 ± 2.72 AB	819 ± 45.4 BC	3391 ± 67.3 BC	182 ± 5.66 ns	685 ± 133 B
1 mM PUT	9.98 ± 0.59 A	27.3 ± 1.98 A	894 ± 57.1 ABC	3599 ± 268 AB	187 ± 12.5 ns	664 ± 222 B
*SD* × *T*						
Before storage	8.19 ± 0.15 i	25.7 ± 0.55 def	852 ± 3.79 e–i	3651 ± 65.5 def	193 ± 9.71 ab	631 ± 49.1 e–i
Day 7 × Control	9.33 ± 0.25 gh	28.0 ± 0.40 abc	1001 ± 23.3 a–e	3699 ± 86.5 cde	188 ± 7.50 ab	717 ± 42.9 d–g
Day 14 × Control	9.67 ± 0.37 d–g	23.3 ± 0.74 ghi	723 ± 49.0 ij	3938 ± 76.2 ab	174 ± 9.29 ab	629 ± 39.5 f–i
Day 21 × Control	10.9 ± 0.17 a	22.3 ± 0.49 hi	592 ± 65.7 j	4038 ± 58.7 a	165 ± 14.0 b	588 ± 14.5 ghi
Day 7 × 0.5 mM CA	9.68 ± 0.34 d–g	25.2 ± 0.59 d–g	1028 ± 52.5 a–d	3514 ± 63.1 efg	182 ± 5.77 ab	780 ± 39.8 de
Day 14 × 0.5 mM CA	9.95 ± 0.01 c–f	26.1 ± 0.72 c–f	895 ± 69.2 b–h	3243 ± 54.1 hi	190 ± 10.9 ab	706 ± 27.8 e–h
Day 21 × 0.5 mM CA	10.9 ± 0.19 a	26.0 ± 0.55 c–f	836 ± 44.6 f–i	3031 ± 52.1 ij	190 ± 9.01 ab	625 ± 26.6 f–i
Day 7 × 1 mM CA	9.40 ± 0.18 gh	25.2 ± 0.70 d–g	967 ± 77.3 a–f	3483 ± 75.1 fg	192 ± 8.73 ab	748 ± 57.4 def
Day 14 × 1 mM CA	9.60 ± 0.19 d–g	26.8 ± 0.83 a–e	1051 ± 50.4 abc	3408 ± 38.6 gh	186 ± 9.64 ab	973 ± 20.1 bc
Day 21 × 1 mM CA	10.1 ± 0.20 cd	27.1 ± 0.35 a–d	1094 ± 29.8 a	3415 ± 65.6 gh	185 ± 7.09 ab	1047 ± 46.1 ab
Day 7 × 0.5 mM SA	10.3 ± 0.11 bc	26.8 ± 0.67 a–e	955 ± 41.1 a–g	3523 ± 63.2 efg	177 ± 12.7 ab	673 ± 65.3 e–h
Day 14 × 0.5 mM SA	9.97 ± 0.13 cde	24.3 ± 0.55 fgh	809 ± 54.0 f–i	2972 ± 74.2 j	181 ± 8.62 ab	973 ± 63.4 bc
Day 21 × 0.5 mM SA	9.75 ± 0.15 d–g	23.3 ± 0.53 ghi	746 ± 40.9 hij	2572 ± 72.1 k	183 ± 9.86 ab	1128 ± 43.4 a
Day 7 × 1 mM SA	8.96 ± 0.12 h	25.5 ± 0.74 def	1057 ± 61.5 ab	3710 ± 41.6 cde	179 ± 11.1 ab	739 ± 55.7 def
Day 14 × 1 mM SA	9.83 ± 0.13 c–g	26.0 ± 0.71 c–f	943 ± 62.1 a–g	3771 ± 56.5 bcd	188 ± 10.0 ab	707 ± 78.6 efg
Day 21 × 1 mM SA	10.1 ± 0.15 cd	26.2 ± 0.32 cde	893 ± 15.3 c–h	3831 ± 82.1 a–d	193 ± 10.7 ab	637 ± 42.5 e–h
Day 7 × 0.5 mM PUT	9.40 ± 0.14 fgh	21.4 ± 0.67 i	842 ± 49.5 e–i	3355 ± 69.6 gh	185 ± 5.29 ab	859 ± 12.1 cd
Day 14 × 0.5 mM PUT	9.67 ± 0.11 d–g	26.5 ± 0.53 b–e	820 ± 43.4 f–i	3396 ± 89.6 gh	182 ± 8.18 ab	613 ± 35.4 f–i
Day 21 × 0.5 mM PUT	9.70 ± 0.10 d–g	26.9 ± 0.51 a–d	795 ± 47.1 ghi	3423 ± 41.4 gh	180 ± 4.04 ab	582 ± 32.9 ghi
Day 7 × 1 mM PUT	10.8 ± 0.10 ab	24.9 ± 0.93 efg	905 ± 53.9 b–h	3276 ± 59.9 h	199 ± 13.7 a	950 ± 67.6 bc
Day 14 × 1 mM PUT	9.70 ± 0.08 d–g	28.4 ± 0.95 ab	895 ± 75.9 c–h	3646 ± 43.9 def	184 ± 6.24 ab	484 ± 36.7 i
Day 21 × 1 mM PUT	9.50 ± 0.08 e–h	28.7 ± 0.57 a	885 ± 63.7 d–i	3876 ± 86.7 abc	178 ± 7.00 ab	557 ± 51.5 hi
*Significant effects* (*p values*)						
SD	0.001	0.729	0.005	0.817	0.305	0.498
T	0.001	0.034	0.001	0.001	0.092	0.001
SD × T	0.001	0.001	0.001	0.001	0.038	0.001

*Note*: Uppercase letters indicate differences in storage duration and treatments. Lowercase letters indicates difference in SD × T interaction.

Abbreviations: ±, standard deviation of mean; Ca, calcium; CA, citric acid; K, potassium; Mg, magnesium; P, phosphorus; PUT, putrescine; SA, salicylic acid.

No significant change was observed in the protein level of broccoli samples during the 21 days of storage. Protein content in 1 mM PUT‐treated broccoli samples was higher than the other treatments. Protein content in the control group decreased by 4.9% compared to before storage. Protein level in broccoli samples 0.5 mM PUT‐treated on 7 days of storage was reduced by 20.1% compared to before storage. Whereas, protein content in broccoli samples 1 mM PUT‐treated on the 21 days of storage increased by 11.7% compared to before storage (Table 4).

Depending on the different SD, fluctuations in mineral composition contents were observed. The most abundant element in broccoli samples was potassium. Depending on the SD, the P content in broccoli samples changed significantly. However, there was no statistical difference in terms of K, Mg, and Ca values with increasing storage duration. When the treatments were evaluated, fluctuations were observed in the element contents. Statistically significant differences were found among treatments in terms of all the element contents examined (except for Mg). The highest contents of P, K, and Ca were determined in the 1 mM CA, control, and 0.5 mM SA treatments, respectively. When assessing the interaction between SD × T, statistically significant differences were observed in relation to all examined element contents. The fluctuations in element contents were observed. The highest levels of P, K, Mg, and Ca were determined at Day 21 × 1 mM CA, Day 21 × Control, Day 7 × 1 mM PUT, and Day 21 × 0.5 mM SA treatments, respectively (Table 4).

### 3.4 Vitamin C, phenolic, and flavonoid changes by storage duration and treatment

SD, T, and SD × T interaction effects were highly significant in vitamin C. Depending on the different SD for broccoli samples stored at 4°C, the highest vitamin C (135 mg 100 g^−1^ FW) was observed before storage, and the lowest vitamin C (115 mg 100 g^−1^ FW) was observed in the broccoli samples after 21 days of storage. When treatments are examined, the highest vitamin C value (129 mg 100 g^−1^ FW) was found in 1 mM CA, and the lowest value (103 mg 100 g^−1^ FW) was observed in the control group. With respect to SD × T interaction, the highest vitamin C were determined before storage (135 mg 100 g^−1^ FW) and at Day 7 × 0.5 mM PUT‐treated broccoli samples (134 mg 100 g^−1^ FW); the lowest vitamin C levels were observed in Day 21 × Control (90.6 mg 100 g^−1^ FW) treatment (Table 5).

**TABLE 5:** Effect of citric acid, salicylic acid, and putrescine on vitamin C, phenolic, and flavonoid contents of broccoli sample during storage at 4°C.

Storage duration (SD)	Vitamin C (mg 100 g^−1^)	Ferulic acid (mg 100 g^−1^)	Gallic acid (mg 100 g^−1^)	Vanillic acid (mg 100 g^−1^)	Chlorogenic acid (mg 100 g^−1^)	Caffeic acid (mg 100 g^−1^)	Quercetin (mg 100 g^−1^)
Before storage	135 ± 1.52 A	2.17 ± 0.06 A	2.40 ± 0.04 AB	2.82 ± 0.05 A	1.03 ± 0.02 A	1.09 ± 0.04 A	1.34 ± 0.04 A
Day 7	130 ± 4.82 A	1.85 ± 0.28 AB	2.33 ± 0.12 A	2.80 ± 0.05 A	0.98 ± 0.06 AB	1.06 ± 0.05 A	1.32 ± 0.04 A
Day14	123 ± 10.8 A	1.71 ± 0.22 BC	2.25 ± 0.18 AB	2.74 ± 0.06 AB	0.95 ± 0.04 B	1.01 ± 0.06 AB	1.29 ± 0.06 A
Day 21	115 ± 11.9 B	1.62 ± 0.25 C	2.15 ± 0.21 B	2.68 ± 0.13 B	0.89 ± 0.04 C	0.98 ± 0.06 B	1.23 ± 0.04 B
*Treatment* (*T*)							
Before storage	135 ± 1.52 A	2.17 ± 0.06 A	2.40 ± 0.04 ABC	2.82 ± 0.05 AB	1.03 ± 0.02 A	1.09 ± 0.04 ns	1.34 ± 0.04 AB
Control	103 ± 13.2 B	1.78 ± 0.34 C	1.92 ± 0.21 E	2.62 ± 0.15 C	0.95 ± 0.07 AB	0.97 ± 0.10 ns	1.24 ± 0.08 BC
0.5 mM CA	128 ± 3.45 A	2.07 ± 0.05 AB	2.41 ± 0.06 AB	2.79 ± 0.03 AB	0.99 ± 0.06 AB	1.05 ± 0.07 ns	1.32 ± 0.04 A
1 mM CA	129 ± 3.32 A	1.83 ± 0.31 ABC	2.42 ± 0.03 A	2.73 ± 0.06 AB	0.94 ± 0.05 AB	1.00 ± 0.05 ns	1.34 ± 0.05 A
0.5 mM SA	120 ± 10.6 A	1.41 ± 0.05 E	2.30 ± 0.10 A–D	2.69 ± 0.06 BC	0.95 ± 0.04 AB	0.97 ± 0.05 ns	1.26 ± 0.04 ABC
1 mM SA	126 ± 4.21 A	1.49 ± 0.05 DE	2.26 ± 0.11 BCD	2.72 ± 0.07 ABC	0.86 ± 0.05 C	1.03 ± 0.05 ns	1.29 ± 0.05 ABC
0.5 mM PUT	127 ± 6.80 A	1.81 ± 0.04 BC	2.14 ± 0.05 D	2.83 ± 0.05 A	0.97 ± 0.05 AB	1.04 ± 0.04 ns	1.22 ± 0.03 C
1 mM PUT	126 ± 6.53 A	1.72 ± 0.04 CD	2.25 ± 0.08 CD	2.78 ± 0.05 AB	0.93 ± 0.02 BC	1.04 ± 0.05 ns	1.28 ± 0.04 ABC
*SD* × *T*							
Before storage	135 ± 1.52 a	2.17 ± 0.05 ab	2.40 ± 0.04 abc	2.82 ± 0.05 abc	1.03 ± 0.02 ab	1.09 ± 0.04 abc	1.34 ± 0.04 ab
Day 7 × Control	120 ± 3.05 hi	2.14 ± 0.05 abc	2.17 ± 0.05 fgh	2.78 ± 0.04 bcd	1.02 ± 0.03 abc	1.11 ± 0.02 a	1.33 ± 0.04 ab
Day 14 × Control	98.0 ± 2.64 k	1.82 ± 0.05 d	1.87 ± 0.04 i	2.64 ± 0.03 fg	0.97 ± 0.03 b–f	0.93 ± 0.02 fg	1.24 ± 0.03 cde
Day 21 × Control	90.6 ± 1.53 L	1.37 ± 0.04 f	1.71 ± 0.04 j	2.43 ± 0.05 h	0.87 ± 0.03 gh	0.88 ± 0.02 g	1.15 ± 0.04 e
Day 7 × 0.5 mM CA	131 ± 1.73 a–d	2.05 ± 0.05 bc	2.46 ± 0.05 a	2.80 ± 0.03 abc	1.06 ± .0.05 a	1.08 ± 0.07 abc	1.34 ± 0.03 ab
Day 14 × 0.5 mM CA	128 ± 2.00 c–f	2.04 ± 0.04 c	2.42 ± 0.04 abc	2.78 ± 0.03 bcd	0.98 ± 0.03 a–d	1.09 ± 0.02 ab	1.35 ± 0.03 ab
Day 21 × 0.5 mM CA	124 ± 2.00 fgh	2.12 ± 0.04 abc	2.35 ± 0.01 a–e	2.79 ± 0.03 abc	0.92 ± 0.03 d–g	0.97 ± 0.05 efg	1.27 ± 0.02 bcd
Day 7 × 1 mM CA	133 ± 2.00 abc	2.22 ± 0.03 a	2.42 ± 0.03 abc	2.80 ± 0.03 abc	0.98 ± 0.03 a–d	1.05 ± 0.05 a–e	1.37 ± 0.02 a
Day 14 × 1 mM CA	129 ± 2.00 a–f	1.72 ± 0.04 d	2.44 ± 0.04 ab	2.75 ± 0.02 be	0.96 ± 0.02 b–f	0.98 ± 0.03 c–g	1.37 ± 0.02 a
Day 21 × 1 mM CA	126 ± 1.53 d–g	1.53 ± 0.05 e	2.40 ± 0.02 abc	2.67 ± 0.04 efg	0.89 ± 0.02 e–h	0.99 ± 0.04 b–f	1.27 ± 0.02 bcd
Day 7 × 0.5 mM SA	130 ± 1.00 a–e	1.44 ± 0.05 ef	2.39 ± 0.03 a–d	2.77 ± 0.03 b–e	0.98 ± 0.04 a–d	1.03 ± 0.03 a–f	1.29 ± 0.03 abc
Day 14 × 0.5 mM SA	124 ± 1.53 e–h	1.37 ± 0.03 f	2.33 ± 0.03 b–e	2.69 ± 0.04 d–g	0.98 ± 0.02 a–d	0.97 ± 0.04 efg	1.27 ± 0.03 bcd
Day 21 × 0.5 mM SA	107 ± 2.07 j	1.41 ± 0.02 ef	2.17 ± 0.06 fgh	2.63 ± 0.03 g	0.91 ± 0.02 d–g	0.93 ± 0.04 fg	1.22 ± 0.01 cde
Day 7 × 1 mM SA	130 ± 1.00 a–e	1.53 ± 0.05 e	2.39 ± 0.03 a–d	2.80 ± 0.02 abc	0.89 ± 0.03 e–h	1.08 ± 0.04 a–d	1.34 ± 0.03 ab
Day 14 × 1 mM SA	127 ± 1.73 def	1.48 ± 0.04 ef	2.25 ± 0.04 efg	2.73 ± 0.03 c–f	0.87 ± 0.05 gh	1.03 ± 0.03 a–f	1.29 ± 0.02 abc
Day 21 × 1 mM SA	121 ± 2.00 ghi	1.45 ± 0.04 ef	2.15 ± 0.05 gh	2.64 ± 0.04 fg	0.82 ± 0.04 h	0.98 ± 0.02 b–g	1.24 ± 0.03 cde
Day 7 × 0.5 mM PUT	134 ± 1.52 ab	1.82 ± 0.04 d	2.17 ± 0.06 fgh	2.88 ± 0.02 a	1.01 ± 0.03 abc	1.00 ± 0.03 b–f	1.24 ± 0.03 cde
Day 14 × 0.5 mM PUT	129 ± 2.08 b–f	1.83 ± 0.03 d	2.16 ± 0.05 gh	2.84 ± 0.03 ab	0.98 ± 0.02 a–d	1.05 ± 0.02 a–e	1.24 ± 0.03 cde
Day 21 × 0.5 mM PUT	119 ± 2.51 hi	1.76 ± 0.03 d	2.10 ± 0.02 h	2.77 ± 0.03 b–e	0.91 ± 0.02 d–g	1.08 ± 0.02 abc	1.20 ± 0.04 de
Day 7 × 1 mM PUT	132 ± 1.73 a–d	1.75 ± 0.04 d	0.32 ± 0.03 cde	2.76 ± 0.04 b–e	0.94 ± 0.02 c–g	1.07 ± 0.04 a–e	1.30 ± 0.03 abc
Day 14 × 1 mM PUT	128 ± 1.15 c–f	1.70 ± 0.02 d	2.28 ± 0.02 def	2.75 ± 0.03 b–e	0.94 ± 0.02 c–g	1.05 ± 0.04 a–e	1.30 ± 0.03 abc
Day 21 × 1 mM PUT	118 ± 2.08 i	1.70 ± 0.03 d	2.16 ± 0.04 fgh	2.83 ± 0.03 ab	0.91 ± 0.02 d–g	1.00 ± 0.02 b–f	1.24 ± 0.03 cde
*Significant effects* (*p values*)							
SD	0.001	0.001	0.001	0.001	0.001	0.001	0.001
T	0.001	0.001	0.001	0.001	0.001	0.063	0.001
SD × T	0.001	0.001	0.001	0.001	0.001	0.001	0.001

*Note*: Uppercase letters indicate differences in storage duration and treatments. Lowercase letters indicates difference in SD × T interaction.

Abbreviations: ±, standard deviation of mean; CA, citric acid; PUT, putrescine; SA, salicylic acid.

SD, T (except for caffeic acid), and SD × T interactions have a highly significant influence on the ferulic acid, gallic acid, vanillic acid, quercetin, caffeic acid, and chlorogenic acid values. The most abundant phenolic content in broccoli samples was determined to be vanillic acid. In comparison to the phenolic and flavonoid values before storage, there was a decrease in these values due to increased SD; in different treatments, fluctuations were observed. Phenolic and flavonoid contents in the control group were higher than in some treatments. The effect of the treatments on the phenolic acid values was also evident. In most cases, the treatments with CA, SA, and PUT tended to result in higher phenolic acid values than the control treatment. However, the effect of the treatments was not consistent across all the phenolic acids. For instance, gallic acid values were higher in the CA‐treated samples than in the control samples. On the other hand, vanillic acid values were higher in the CA and PUT‐treated samples compared to the control. CA treatment showed a positive effect on most of the phenolic acids, except for chlorogenic acid, which showed a decreasing trend with increasing CA concentration. SA treatment also showed a positive effect on most of the phenolic acids, except for ferulic acid and caffeic acid. PUT treatment generally exhibited a positive effect on the phenolic acid values. The highest flavonoid (quercetin) contents were determined in the 0.5 and 1.0 mM CA treatments, while the lowest value was observed in the 0.5 mM PUT treatment (Table 5).

### 3.5 Biochemical change by storage duration and treatment

SD had highly significant effects on the proline, total carbohydrate, glucose, and sucrose contents. However, SD did not have a significant effect on anthocyanin and free amino acids. In addition, the T and SD × T interactions had a highly significant effect on all the properties examined. The anthocyanin concentration exhibited a gradual decline over the SD, reaching its lowest point at 21 days. Proline content displayed fluctuations, initially increasing at 7 days, followed by a slight decrease at 14 and 21 days. Free amino acid content generally increased throughout the storage, peaking at 14 days. Total carbohydrate and glucose contents fluctuated over the storage, while sucrose content generally increased and reached its highest level at 21 days. As seen in Table 6, anthocyanin content increased in the 1 mM PUT treatment compared with the control, while anthocyanin levels decreased in the other treatments compared to the control. The treatments with the highest proline levels were 1 mM CA and 1 mM PUT, whereas the lowest contents were seen in the 1 mM SA and 0.5 mM PUT treatments. Compared with the control group, the levels of free amino acids increased in broccoli samples treated with 0.5 mM and 1 mM CA, as well as 1 mM PUT. Conversely, the content of free amino acids decreased in samples treated with 0.5 mM SA, 1 mM SA, and 0.5 mM PUT. The highest total carbohydrate and glucose contents were determined in the 1 mM SA and control treatments. However, 0.5 mM PUT‐treated broccoli samples possessed the lowest total carbohydrate and glucose contents. The highest sucrose contents were detected in the 1 mM CA and 1 mM PUT treatments. Higher sucrose values were obtained from all treatments in the study compared to the control.

**TABLE 6:** Effect of citric acid, salicylic acid, and putrescine on biochemical contents of broccoli sample during storage at 4°C.

Storage duration (SD)	Anthocyanin (μmol mL^−1^)	Proline (mg 100 g^−1^)	Free amino acid (mg 100 g^−1^)	Total carbohydrate (mg 100 g^−1^)	Glucose (mg 100 g^−1^)	Sucrose (mg 100 g^−1^)
Before storage	2.45 ± 0.02 ns	174 ± 0.54 B	50.1 ± 0.02 ns	512 ± 11.5 AB	76.9 ± 0.12 AB	20.1 ± 0.29 B
Day 7	2.20 ± 0.24 ns	183 ± 10.8 A	52.9 ± 4.18 ns	487 ± 36.8 B	73.1 ± 5.52 B	26.0 ± 4.33 A
Day14	2.09 ± 0.31 ns	181 ± 12.8 AB	55.1 ± 5.37 ns	533 ± 38.6 A	80.1 ± 5.72 A	25.1 ± 4.13 AB
Day 21	2.07 ± 0.22 ns	173 ± 9.43 B	54.6 ± 3.27 ns	483 ± 30.0 B	72.5 ± 4.50 B	27.6 ± 0.91 A
*Treatment* (*T*)						
Before storage	2.45 ± 0.02 AB	174 ± 0.55 ABC	50.1 ± 0.02 C	512 ± 11.5 ABC	76.9 ± 0.12 ABC	20.1 ± 0.29 C
Control	2.32 ± 0.16 AB	172 ± 2.10 BC	51.8 ± 3.57 C	533 ± 38.9 A	80.0 ± 5.85 A	19.7 ± 5.24 C
0.5 mM CA	2.07 ± 0.16 CD	182 ± 8.17 AB	58.1 ± 3.80 AB	489 ± 32.9 ABC	73.4 ± 4.94 ABC	27.6 ± 0.37 AB
1 mM CA	1.90 ± 0.08 DE	190 ± 4.34 A	54.7 ± 2.21 BC	500 ± 29.1 ABC	75.1 ± 4.36 ABC	28.5 ± 0.36 A
0.5 mM SA	1.80 ± 0.12 E	184 ± 15.6 AB	51.7 ± 1.77 C	479 ± 34.6 BC	71.9 ± 5.18 ABC	25.0 ± 1.6 B
1 mM SA	2.07 ± 0.24 CD	167 ± 7.33 C	51.7 ± 1.98 C	534 ± 15.8 A	80.1 ± 2.37 A	27.7 ± 1.25 AB
0.5 mM PUT	2.21 ± 0.03 BC	169 ± 2.43 C	51.7 ± 2.13 C	455 ± 6.15 C	68.3 ± 0.90 C	26.6 ± 0.80 AB
1 mM PUT	2.50 ± 0.08 A	189 ± 7.12 A	59.9 ± 4.81 A	518 ± 52.9 AB	77.7 ± 7.94 AB	28.7 ± 1.12 A
*SD* × *T*						
Before storage	2.45 ± 0.02 bc	174 ± 0.55 de	50.1 ± 0.02 g	512 ± 11.5 fg	76.9 ± 0.12 fg	20.1 ± 0.29 j
Day 7 × Control	2.51 ± 0.01 b	173 ± 1.36 de	49.7 ± 0.18 g	553 ± 2.90 c	83.0 ± 0.44 c	16.6 ± 0.98 k
Day 14 × Control	2.32 ± 0.03 d	174 ± 1.58 de	49.1 ± 0.21 g	565 ± 3.84 b	84.8 ± 0.58 b	15.9 ± 0.47 k
Day 21 × Control	2.15 ± 0.02 g	169 ± 2.11 def	56.5 ± 0.17 d	482 ± 2.92 h	72.3 ± 0.44 h	26.6 ± 0.12 efg
Day 7 × 0.5 mM CA	2.18 ± 0.01 fg	187 ± 1.89 ab	53.0 ± 0.12 e	478 ± 3.57 h	71.8 ± 0.54 h	27.6 ± 0.17 de
Day 14 × 0.5 mM CA	2.19 ± 0.02 efg	186 ± 2.35 ab	60.2 ± 0.15 c	531 ± 2.72 d	79.7 ± 0.41 d	28.1 ± 0.13 bcd
Day 21 × 0.5 mM CA	1.85 ± 0.02 i	170 ± 0.67 def	60.9 ± 0.18 bc	458 ± 3.79 ijk	68.7 ± 0.57 ijk	27.3 ± 0.11 def
Day 7 × 1 mM CA	1.82 ± 0.02 ij	193 ± 2.31 ab	53.4 ± 0.22 e	503 ± 5.16 g	75.4 ± 0.77 g	28.7 ± 0.10 bc
Day 14 × 1 mM CA	1.88 ± 0.01 i	193 ± 0.89 ab	57.6 ± 0.16 d	532 ± 157 d	79.9 ± 0.23 d	28.1 ± 0.17 bcd
Day 21 × 1 mM CA	1.99 ± 0.03 h	185 ± 1.21 bc	53.0 ± 0.20 e	466 ± 2.00 i	69.9 ± 0.30 i	28.8 ± 0.22 bc
Day 7 × 0.5 mM SA	1.96 ± 0.02 h	195 ± 3.11 a	53.8 ± 0.18 e	454 ± 4.28 jk	68.0 ± 0.64 jk	24.8 ± 0.31 h
Day 14 × 0.5 mM SA	1.71 ± 0.02 k	193 ± 1.45 ab	51.5 ± 0.17 f	526 ± 4.28 de	78.8 ± 0.64 de	23.8 ± 0.12 i
Day 21 × 0.5 mM SA	1.74 ± 0.01 k	163 ± 2.98 fg	49.7 ± 0.18 g	459 ± 2.36 ijk	68.9 ± 0.35 ijk	26.3 ± 0.28 fg
Day 7 × 1 mM SA	2.26 ± 0.04 de	176 ± 3.11 cd	49.5 ± 0.17 g	516 ± 2.90 ef	77.3 ± 0.44 ef	29.0 ± 0.21 b
Day 14 × 1 mM SA	1.75 ± 0.02 jk	160 ± 1.23 g	51.4 ± 0.18 f	551 ± 4.52 c	82.7 ± 0.68 c	26.1 ± 0.16 g
Day 21 × 1 mM SA	2.19 ± 0.01 efg	163 ± 1.47 fg	54.0 ± 0.11 e	534 ± 3.57 d	80.1 ± 0.54 d	27.9 ± 0.18 cd
Day 7 × 0.5 mM PUT	2.22 ± 0.03 ef	167 ± 2.56 efg	49.4 ± 0.25 g	450 ± 2.69 k	67.5 ± 0.40 k	25.8 ± 0.14 g
Day 14 × 0.5 mM PUT	2.23 ± 0.03 ef	169 ± 1.00 d–g	51.3 ± 1.52 f	453 ± 1.53 k	68.1 ± 0.20 jk	26.5 ± 0.33 fg
Day 21 × 0.5 mM PUT	2.20 ± 0.03 efg	171 ± 3.03 def	53.9 ± 0.15 e	463 ± 2.48 ij	69.4 ± 0.37 ij	27.5 ± 0.23 de
Day 7 × 1 mM PUT	2.47 ± 0.03 bc	187 ± 12.3 ab	61.9 ± 0.17 b	456 ± 1.78 jk	68.3 ± 0.27 jk	30.0 ± 0.24 a
Day 14 × 1 mM PUT	2.60 ± 0.03 a	192 ± 2.11 ab	64.1 ± 0.28 a	578 ± 2.72 a	86.6 ± 0.41 a	27.4 ± 0.19 de
Day 21 × 1 mM PUT	2.41 ± 0.04 c	188 ± 4.71 ab	53.6 ± 0.23 e	521 ± 3.34 ef	78.2 ± 0.50 ef	28.7 ± 0.35 bc
*Significant effects* (*p values*)						
SD	0.062	0.023	0.164	0.001	0.001	0.004
T	0.001	0.001	0.001	0.001	0.001	0.001
SD × T	0.001	0.001	0.001	0.001	0.001	0.001

*Note*: Uppercase letters indicate differences in storage duration and treatments. Lowercase letters indicates difference in SD × T interaction.

Abbreviations: ±, standard deviation of mean; CA, citric acid; PUT, putrescine; SA, salicylic acid.

There is no consistent trend for the effect of SD × T on anthocyanin levels. The anthocyanin content of broccoli samples ranged from 1.71 to 2.60 μmol mL^−1^ DW, and the highest value was observed in Day 14 × 1 mM PUT treatment. The levels of proline seem to be relatively stable across the different treatments and storage durations. The highest proline content was found at Day 7 × 0.5 mM SA treatment. The free amino acid content of broccoli samples varied from 49.1 (Day 14 × Control) to 64.1 (Day 14 × 1 mM PUT) mg 100 g^−1^ DW. The total carbohydrate content of broccoli samples ranged from 450 to 578 mg 100 g^−1^ DW, and the highest value was observed in Day 14 × 1 mM PUT‐treated broccoli samples. Glucose and sucrose were the two most abundant individual carbohydrates in broccoli samples, and their contents ranged from 67.5 to 86.6 mg 100 g^−1^ DW and from 15.9 to 30.0 mg 100 g^−1^ DW, respectively. The highest glucose and sucrose contents were found in Day 14 × 1 mM PUT and Day 7 × 1 mM PUT treatments, respectively (Table 6).

### 3.6 Correlation and heatmap analysis

The purpose of correlation analysis is to reveal a positive or negative relationship between the examined features. In this context, it was investigated whether there were relationships between the examined properties depending on the storage period and the treatments applied.

**FIGURE 1:**
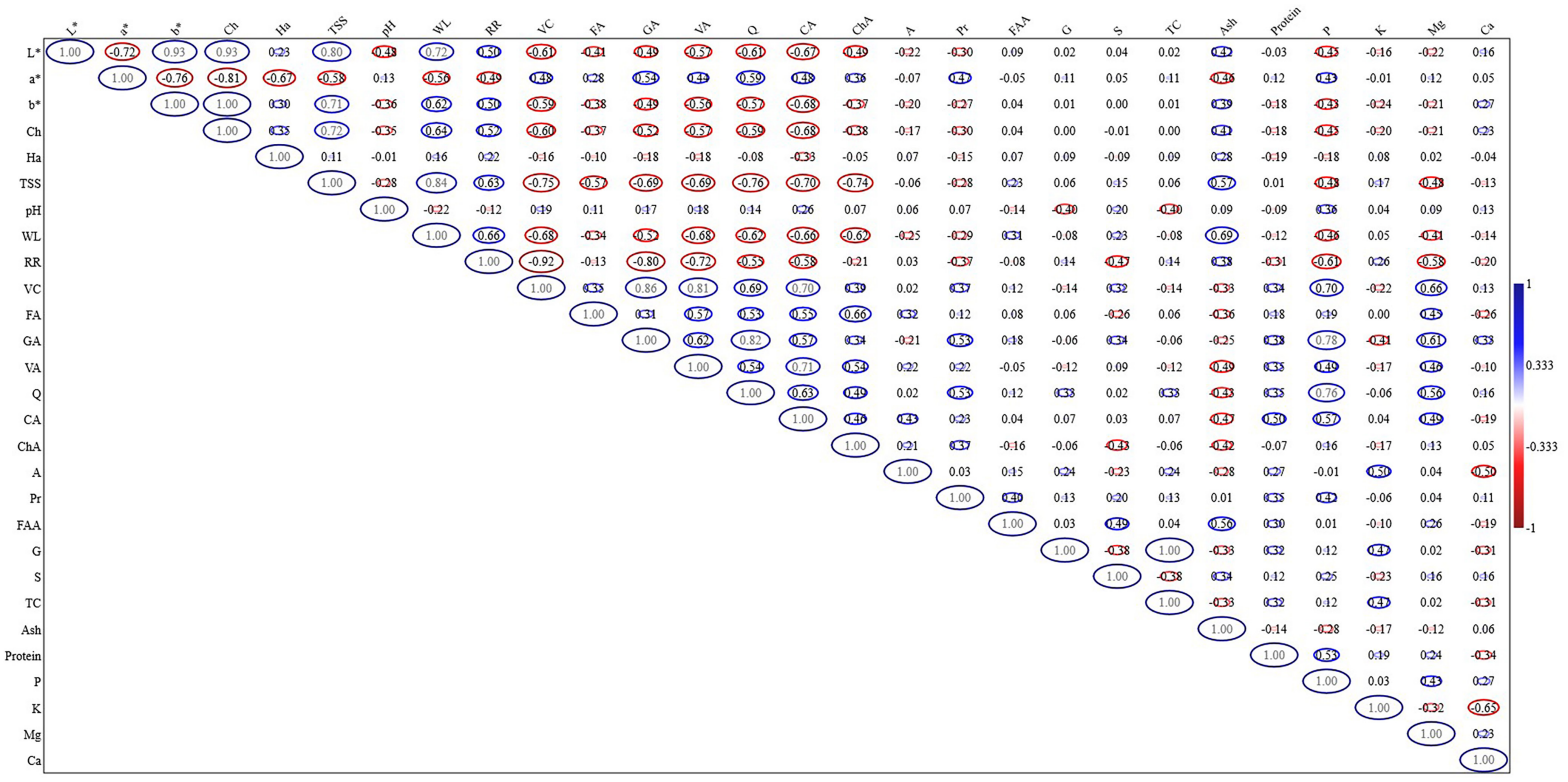
Correlation matrix of examined properties depending on different treatments during storage at 4°C, where +1.0 and −1.0 represent stronger positive and negative correlations between the two properties, respectively. WL, weight loss; RR, respiratory rate; TSS, total soluble solids; Ch, chroma; Ha, hue angle; P, phosphorus; K, potassium; Mg, magnesium; Ca, calcium; VC, vitamin C; FA, ferulic acid; GA, gallic acid; VA, vanillic acid; Q, quercetin; CA, caffeic acid; ChA, chlorogenic acid; A, anthocyanin; Pr, proline; FAA, free amino acid; TC, total carbohydrate; G, glucose; S, sucrose.

WL exhibited positive associations with TSS (*r* = 0.84), ash (*r* = 0.69), RR (*r* = 0.66) and L* (*r* = 0.65), while WL exhibited negative associations with vanillic acid (*r* = −0.68) and vitamin C (*r* = −0.68). RR showed a highly significant negative correlation with vitamin C (*r* = −0.92). TSS enclosed significant positive correlations with L* (*r* = 0.80), chroma (*r* = 0.72), and b* (*r* = 0.71) color properties, except for a*. TSS enclosed significant negative correlations with phenolic and flavonoid contents. While the pH value had significant negative correlations with the L* (*r* = −0.49) value, it did not correlate with all the other traits examined. Chroma value had significant positive correlations with b* (*r* = 1.00) and L* (*r* = 0.93) values, while it had significant negative correlations with a* (*r* = −0.81), caffeic acid (*r* = −0.68), vitamin C (*r* = −0.65), and quercetin (*r* = −0.59). K content had a significant negative correlation with Ca (*r* = −0.50) content, it had a significant positive correlations with anthocyanin (*r* = 0.50) content. Gallic acid showed the highest positive correlation with quercetin (*r* = 0.82) and P (*r* = 0.78). Vitamin C showed the highest positive correlations with gallic acid (*r* = 0.86) and vanillic acid (*r* = 0.81) contents (Figure 1).

**FIGURE 2:**
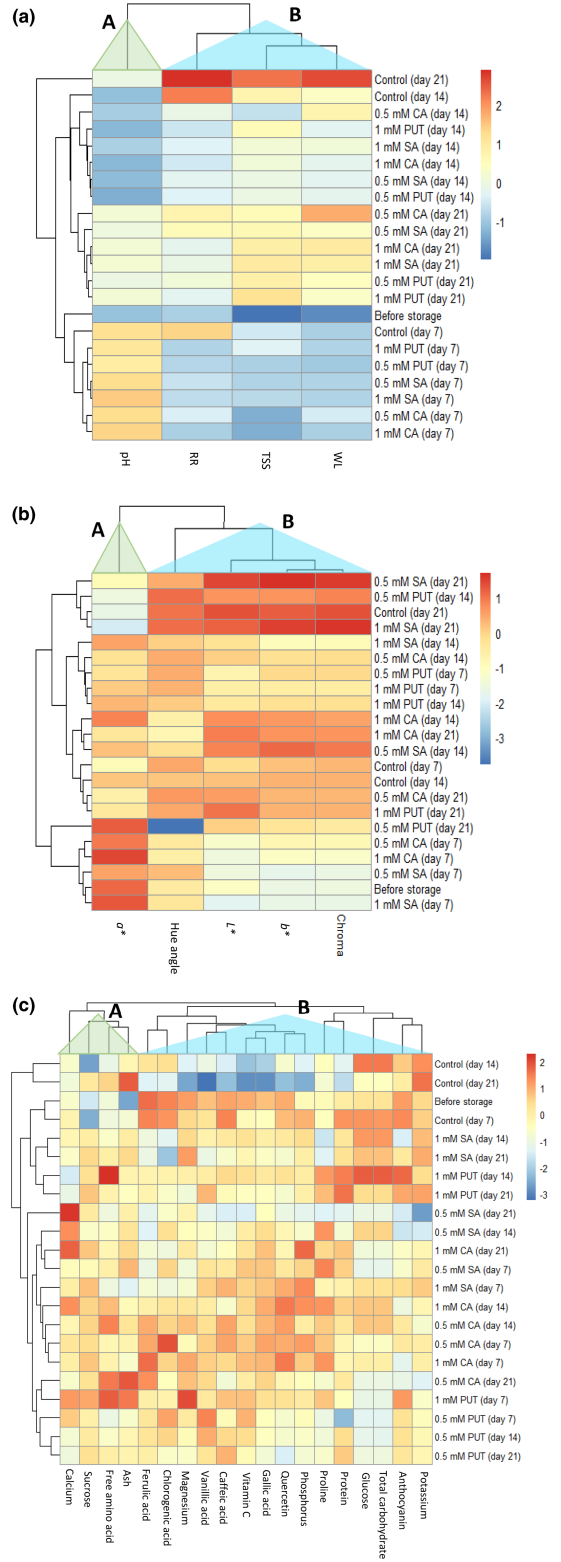
Changes of the ‘Marathon F_1_’ broccoli sample during cold storage. Heatmap of weight loss (WL), respiration rate (RR), total soluble solids (TSS), and pH values in different treatments (a) and color properties (b). Heatmap of proximate and mineral composition, vitamin C, phenolic, flavonoid, and biochemical contents in different treatments (c).

Hierarchical clustering categorized the variables into two clusters, namely, Cluster A and Cluster B, as illustrated in Figure 2. While the pH variable of cluster A decreased in control (14 days), 0.5 mM CA (14 days), 1 mM PUT (14 days), 1 mM SA (14 days), 1 mM CA (14 days), 0.5 mM SA (14 days), and 0.5 mM PUT (14 days), it increased in other treatments. Whereas, these parameters are lowest in control (7 days), 1 mM PUT (7 days), 0.5 mM PUT (7 days), 0.5 mM SA (7 days), 1 mM SA (7 days), 0.5 mM CA (7 days), and 1 mM CA (7 days) treatments. When examining cluster‐B, respiratory rate, soluble solids content, and weight loss showed the highest increase in control (21 days) application. While these three parameters gave good results in the 7th d treatments, they were adversely affected by the increase in storage duration (Figure 2a).

The a* color variable was grouped in cluster‐A, and the L*, b*, chroma and hue angle variables were grouped in cluster‐B. While a* color variable showed significant positive correlations with 1 mM SA (14 days), 1 mM CA (14 days), 0.5 mM PUT (21 days), 0.5 mM CA (7 days), 1 mM CA (7 days), 0.5 mM SA (21 days), before storage, and 1 mM SA (7 days) treatments, it showed a negative correlation with other treatments. The hue angle in cluster B showed the highest negative correlation with the 0.5 mM PUT (21 days) treatment. However, it exhibited the strongest positive correlation with 0.5 mM PUT (14 days), the control group (21 days), and 1 mM SA (21 days). The greatest effect on chroma value occurred in the 0.5 mM SA (21 days) and 1 mM SA (21 days) treatments (Figure 2b).

As shown in Figure 2c, the phenolic, flavonoid, and biochemical contents detected in this study clustered into two main groups in the heatmap dendrogram: cluster‐A, consisting of four contents (calcium, sucrose, free amino acid, and ash), and all other features examined are included in cluster‐B. Calcium content was lowest in the 1 mM PUT (14 days) treatment. The sucrose content was the lowest in the control (day 14) application. Magnesium, vanillic acid, caffeic acid, vitamin C, gallic acid, quercetin, and phosphorus contents were found in the lowest control (21 days) application, and the highest decrease occurred in these contents. The most adverse effects on protein were observed in the control (14 days), control (21 days), 0.5 mM SA (21 days), and 0.5 mM PUT (7 days) treatments. 0.5 mM SA (21 days) treatment did not show a positive effect on potassium content.

## 4 DISCUSSION

Vegetables are especially indefensible to quick water loss (Kays, 1991). The reduction in moisture content leads to a decline in the overall weight and a loss of freshness, appearance, and texture. WL during the storage of vegetables is considered as a quality‐decrease parameter. In addition to the fact that broccoli samples have a texture that cannot prevent high transpiration rate, a high respiration rate can cause an increase in WL during storage and a short shelf life. Depending on the SD × T interaction, an increase occurred with increasing storage duration in WL. The lowest increase in the evaluation made for 21 days was observed in the 0.5 mM SA treatment. The losses increased in all treatments and in the control group with advancing storage periods. However, the greatest losses were observed within the control group, indicating that the treatments effectively decelerated the rate of weight loss increase. Barth et al. (1993) and Serrano et al. (2006) also reported similar findings in broccoli stored under different conditions. In the study by Alali et al. (2023), it was observed that citric acid has the ability to close stomata, decrease transpiration rates, and mitigate weight loss in fruits and vegetables. Similarly, research conducted by Eleni and Theodoros (2011), Babu et al. (2014) and Rastegar et al. (2022) highlighted the anti‐aging properties of CA, SA, and PUT, which also proved beneficial in reducing weight loss in vegetables by scavenging reactive oxygen species and preserving membrane integrity. Mustafa et al. (2018a) reported that SA treatment is important for the degree of inhibition of the metabolic activity of the fruit and that 1 mM SA treatment delayed weight loss in mangosteen after 14 days of storage. Our own investigation further supports these findings, affirming that treatments involving CA, SA, and PUT are effective in reducing weight loss in broccoli samples.

The reduction of RR in broccoli samples was most pronounced after 21 days of storage for all treatments involving CA, SA, and PUT compared to untreated samples. The decline in RR was directly proportional to the concentrations of CA, SA, and PUT, gradually diminishing as these concentrations increased. Thus, it can be said that the decrease in RR with CA, SA, and PUT treatment delays deterioration. The suppression of RR with CA, SA, and PUT treatment has also been reported earlier in numerous vegetables and fruits, including sweet pepper (Rao et al., 2011), mango (Razzaq et al., 2013), green pepper (Dobón‐Suárez et al., 2021), and pear (Adhikary et al., 2021). Srivastava and Dwivedi (2000) reported that the SA treatment caused a reduction in RR depending on concentration. Again, RR was found to be lower in increased CA (0.5 and 1 mM) and SA (0.5 and 1.0 mM) applications in menthe and sage fresh‐cut herbs stored at 5°C for 5 days (Abdel‐Hamid, 2020). It has been observed that exogenous applications slow down the RR in fruit and vegetables. In our study, it was revealed that CA, SA, and PUT treatments slowed down the RR during storage due to the reduction of the amount of carbon dioxide released as a result of respiration.

The TSS values of the samples exhibited an increase, which was influenced by the elevated concentrations of CA, SA, and PUT. However, this increase was less pronounced when compared to the control applications. The rise in TSS levels during storage might be attributed to WL, a phenomenon reported by Moreno et al. (2008), who noted that both dehydration and TSS degradation during storage contribute to WL. It is worth noting that CA, SA, and PUT concentrations possess the potential to reduce transpiration and respiration. This is achieved by regulating cell wall degradation and slowing down ethylene biosynthesis, as highlighted by Alali et al. (2023) and Chen et al. (2023). Sucrose biosynthesis, accelerated by ethylene‐induced sucrose phosphate synthase enzyme action during storage, leads to an increase in TSS (Singh et al., 2019). Similar results reported that CA, SA, and PUT treatments caused major changes in TSS during storage. In a study by Ahmad and Ali (2019), it was observed that TSS in carambola fruits treated with PUT was lower compared to those in the control group, indicating that SA impeded starch degradation. In a separate investigation conducted by Davarynejad et al. (2015), they explored the impact of SA treatment (4 mM) on the postharvest longevity and physiological characteristics of ‘Santa Rosa’ plums. The findings revealed that the SA‐treated plums exhibited the lowest TSS levels, while the control group had the highest TSS levels throughout the storage period. It can be concluded that the treatments applied in our study, similar to other studies, had a slowing effect on the increase in TSS content.

The pH variation observed during storage is linked to the proliferation of microorganisms and the subsequent generation of organic acids, as noted by Heard (2002). pH contents fluctuated depending on storage duration and treatments. Generally, pH contents decreased on the 14th day of storage and increased on the 21st day. Similar findings were stated for apricot and plum, and peach in studies conducted by Zokaee Khosroshahi and Esna‐Ashari (2008) and Davarynejad et al. (2015). A contrary situation to our study was determined by Banda et al. (2014). It has been stated that the pH content of the pomegranate did not change depending on the applied CA after 5 days of storage at 5°C. In our study, the data indicated that the concentrations of PUT were more effective than the CA and SA concentrations in changing pH contents during storage. Şahin (2022) conducted a study examining how various doses of CA, SA, and PUT treatments affected the pH levels of *P. ostreatus* mushrooms following a 14‐day storage period. As a result, it was reported that pH contents increased compared to the control at the end of 7 and 14 days depending on all three treatments. In our study, it was determined that the pH contents increased at the end of the 7 days compared to the control, except for 0.5 mM and 1 mM PUT‐treated, and decreased on the 21 days. However, after 14 days of storage, fluctuations emerged as an increase and decrease. The results we obtained were similar to the results of Şahin (2022), except for PUT‐treated. pH has an important effect on pigments (e.g., chlorophyll, carotenoids, anthocyanins, etc.) responsible for the color of vegetables. Thus, knowledge of pH is necessary to produce safe, high‐quality, and value‐added products (Andrés‐Bello et al., 2013). Consumers are interested in colored‐food products due to the aesthetic, nutritional, and safety aspects of foods, which have increased the demand for natural pigments such as betacyanins, including betanin, anthocyanins, carotenoids, and chlorophylls (Sarker & Oba, 2021).

The color of fresh vegetables is a significant organoleptic factor because color influences consumers' assessments of the product. When the color became darker, a decrease in L* and hue angle were observed. The hue angle represents true color, which is effective for visualizing the color view of vegetables and fruit (Eleni & Theodoros, 2011). Changes in color contents were observed depending on SD and T. Compared with the control, the positive and negative effects of CA, SA, and PUT applications on color were revealed. Depending on the SD × T interaction, the L*, a*, b*, chroma, and hue angle values in the control application on the 21 days of storage gave better results than the 0.5 mM CA, 1 mM CA, 0.5 mM SA, 1 mM SA, 0.5 mM PUT, and 1 mM PUT treatments. However, the positive effects of CA, SA, and PUT treatments on color were revealed after 7 and 14 days of storage. The increase in b* values on the 21st day can be considered as a sign of yellowing in broccoli samples (Table 3). Similarly, postharvest SA and PUT applications retarded color development in different fruits (Ahmad & Ali, 2019; Banda et al., 2014; Koyuncu et al., 2019; Malik et al., 2003). CA has been stated for its inhibitory activity on PPO (Langdon, 1987) and its antibrowning activity in processed fruit and vegetables (Ahvenainen, 1996). In our research, we found that applying CA resulted in a delay in color change, consistent with previous studies. The essential attributes, choice, preference, and acceptability of consumers are broadly and significantly influenced by color attributes, tests, and the nutritional quality of vegetables. Across them, color is the most vital indicator for evaluating the biochemical composition of vegetables (Sarker & Oba, 2021).

The ash content of vegetables is typically composed of various minerals, such as calcium, potassium, magnesium, and phosphorus. These minerals play a crucial role in a range of physiological processes within the human body, including the maintenance of healthy bones, proper nerve function, and optimal muscle performance. The findings reveal that the ash content in samples treated with 0.5 mM CA (10.2% ± 0.62) and 1 mM CA (9.69% ± 0.35) was significantly elevated compared to untreated samples (9.97% ± 0.76). CA is known to chelate metal ions and enhance their solubility, which could have resulted in a higher ash content due to the increased availability of minerals in the product (Sánchez‐Moreno et al., 1998). Additionally, previous studies have reported an increase in ash content in horticulture products‐treated with CA due to the enhanced activity of acid phosphatase and phytase enzymes that release phosphorus from phytate, resulting in a higher ash content. The samples treated with SA and PUT did not exhibit a significant difference in ash content compared to the untreated samples. Ozturk et al. (2021) stated that significant increases were observed in the amount of ash as a natural result of water loss with the progression of the storage period in *Cantharellus cibarius* mushrooms stored at 0°C for 12 days. Researchers also reported that the ash content at the end of the storage period was lower in the CA application than in the control. The increase in ash content with storage duration is likely due to the loss of moisture, leading to a higher concentration of minerals in the remaining product. The increase in ash content in products treated with CA could be attributed to the enhanced solubility of minerals and the release of phosphorus from phytate. Broccoli is a rich source in terms of minerals and phytochemicals (Serrano et al., 2006; Xua et al., 2021). Vallejo et al. (2003) reported substantial decreases in the levels of diverse minerals and bioactive compounds in broccoli post‐harvest, even when stored in cold conditions. In the study, the P, K, Mg, and Ca contents of the CA, SA, and PUT treatments changed depending on the 21‐day storage period. Compared to the control, these changes appeared to occur as increases or decreases. At 7, 14, and 21 days of storage, P (except 7 and 14 days), K, and Mg contents decreased in general, while Ca contents increased. Several prior research investigations have proposed that K acts as an osmotic agent, maintaining a water potential gradient. This, in turn, helps in preserving cell expansion and the overall freshness of biological cells, as discussed by Mpelasoka et al. (2003), Keller and Shrestha (2014), and Guo et al. (2018). Concurrently, P plays a crucial role in sustaining cell metabolism and the vibrant green coloration of fruits and vegetables, as highlighted by Tran et al. (2010) and Guo et al. (2018). Motamedi et al. (2020) reported that the application of polyamine in the postharvest period had a greater effect on the nutritional value of *A. bisporus* mushroom and increased its nutritional value. The heightened absorption of nitrogen, P, and K minerals resulting from polyamine application is thought to be a consequence of polyamines influencing various biochemical and physiological processes. In studies on *P. ostreatus* (Ventura‐Aguilar et al., 2017) and *A. bisporus* (Gupta & Bhat, 2016) mushroom species, it was determined that postharvest CA applications had a positive effect on extending the shelf life and preserving postharvest quality. Jia et al. (2018) reported that postharvest PUT treatment is a promising method for maintaining postharvest quality in cucumber. Üner (2021), in her study on parsley, stated that postharvest SA applications help maintain the quality. In the current study, examined mineral contents were tried to be protected with CA, SA, and PUT treatments. Treatments were found to be generally effective compared with control.

Protein is an essential macronutrient that plays a crucial role in human nutrition, as it provides the building blocks for tissues, enzymes, hormones, and antibodies. The protein content of fruit and vegetables varies widely depending on the type of plant, the stage of maturity, and the environmental conditions during growth. Overall, the results suggest that the effect of different treatments on protein content in broccoli during storage is complex and time‐dependent. While treatments like CA, SA, and PUT can enhance protein content during the initial days of storage, their effect on protein content changes as the storage period progresses. The complex interactions between these treatments and the various metabolic pathways involved in protein synthesis and degradation require further investigation to fully understand the underlying mechanisms. Tarafder et al. (2023) reported that the protein content of broccoli after storage at 4°C varied between 1.87% and 3.49%, depending on different packaging practices. These results are lower than the results we obtained in our study. The reason for this difference can be explained by genotype, growing conditions, and different packaging conditions applied.

Vitamin C, a key bioactive compound, is susceptible to changes during processing and preservation. The findings of this study demonstrated that treatments with CA, SA, and PUT effectively preserved vitamin C during storage, outperforming the control group. It appears that there is a general trend of decreasing vitamin C content over time, with the samples stored for 21 days showing the lowest vitamin C levels compared to those stored for 7 or 14 days. This decrease in vitamin C over time could be due to oxidation or enzymatic degradation, which are both processes that can occur during storage. Tarafder et al. (2023) reported that the Vitamin C content of broccoli varied between 53.01 and 78.41 mg 100 g^−1^ as a result of storage at 4°C. These results are lower than the results we obtained in our study. The reason for this difference can be explained by genotype, growing conditions, and different storage conditions applied. Maketup and Krajayklang (2016) reported that vitamin C in fresh‐cut pineapple dipped in CA was also reduced during storage. However, they stated that this decrease was less than the control group samples. Vitamin C is an antioxidant, which means that it can react with oxygen and get oxidized. This reaction can cause a decrease in vitamin C content. The longer vegetables are stored, the more their vitamin C content will degrade. In our investigation, it was observed that vitamin C levels declined as storage duration increased, and the rate of this decline was mitigated when exogenous treatments of CA, SA, and PUT were administered. Furthermore, regardless of the treatments applied, there was a consistent downward trend in phenolic and flavonoid contents as the storage duration extended. Notably, the control group exhibited higher levels of phenolic and flavonoid contents compared to certain treatment groups. Vanillic acid was determined to be the most abundant phenolic acid in broccoli samples. It was followed by gallic acid and ferulic acid, respectively. The rapid decline in phenolic and flavonoid levels observed in the control group may be attributed to changes in membrane permeability and cell structure deterioration as broccoli enters the senescence phase. The ability of CA, SA, and PUT treatments to maintain phenolic and flavonoid levels can be attributed to their capacity to delay the senescence process in broccoli samples. Consequently, PPO comes into contact with phenolic compounds, leading to their oxidation. The primary reason for the higher retention of phenolic and flavonoid levels in response to CA, SA, and PUT treatments is the delayed senescence and reduced PPO activity (Alali et al., 2023; Rastegar et al., 2022; Razzaq et al., 2013). However, more investigation is needed to illuminate these biochemical processes in different broccoli cultivars. Our findings demonstrate that administering CA, SA, and PUT to broccoli preserves its quality by preventing the degradation of essential bioactive compounds such as vitamin C, phenolic compounds, and flavonoids.

The study's findings revealed that the anthocyanin levels in broccoli samples were particularly influenced by treatments of 1 mM CA and 0.5 mm SA. Guo et al. (2014) reported that anthocyanin contents in CL80, HLZS, and LY broccoli sprouts were approximately 10 mg 100 g^−1^ FW. The results obtained from our study were found to be lower than this value. It can be said that variety, spraying, fertilization, and environmental conditions are effective in making this difference. Jones (2007) reported that anthocyanin content tends to increase after harvest. However, it was detected that the increasing trend was prevented by the treatments in this study. On the flip side, the proline level exhibited fluctuations over the course of 21 days of storage. The highest proline content occurred on Day 21 × 0.5 mM SA‐treated. Malekzadeh et al. (2023) emphasized that high proline content was effective in preserving the green color of broccoli. It was observed that the treatments with high proline content determined in this study preserved the green color in broccoli samples. The reduction of glucose and sucrose values in stored broccoli samples can be related to its use by the plant to generate the energy required for its metabolism, making sugars the main substrate in the respiration duration (Loredana et al., 2023; Pinela et al., 2016). They serve as pioneers for the biosynthesis of polysaccharides, lipids, proteins, and other compounds (Duffus & Duffus, 1984; Parker, 2020). Previous studies showed glucose to be more abundant than sucrose in broccoli (Hasperué et al., 2014). In this study, glucose was found to be the most abundant type of sugar, followed by sucrose. Similarly, previous studies have found glucose to be the main sugar in different broccoli varieties (Bhandari & Kwak, 2015; King & Morris, 1994). Sugars play a crucial role in both the quality and preservation of broccoli samples, serving as the primary source of energy for numerous chemical reactions responsible for synthesizing new compounds and maintaining the integrity of tissues (Hasperué et al., 2014). Several studies have examined the relationship between sugar content and the rate of senescence in broccoli samples (Nishikawa et al., 2005). Given that sugar serves as a vital energy source and a key substrate for respiration, the sugar content is closely linked to the physicochemical characteristics of horticultural crops (Tian et al., 2016). Since broccoli is harvested while still in its immature state, it requires a continuous supply of energy to support high respiration rates (King & Morris, 1994). Sucrose, being one of the principal compounds involved in metabolic reactions, undergoes a 50% reduction in broccoli samples stored at room temperature within 6 h of harvest (Parker, 2020). The postharvest decline in sugar levels is influenced by the rate of respiration, and higher storage temperatures lead to increased respiration rates in broccoli samples (Tian et al., 2016; Parker, 2020). In this study, the storage temperature is constant. However, higher glucose and sucrose contents were found in the 1 mM SA and 1 mM PUT treatments. It has been revealed that 1 mM treatments do not preserve the sugar content but increase the sugar content. Therefore, it can be said that 1 mM treatments preserve the sugar content better.

Correlation analysis is a statistical technique used to measure the strength and direction of a linear relationship between two quantitative variables. In simpler terms, it helps us understand how changes in one variable are associated with changes in another. In this context, it was investigated whether there were relationships between the examined properties depending on the storage period and the treatments applied. It was also revealed by correlation analysis that there was an increase in TSS content due to the increase in WL, which is one of the important quality indicators. The increase occurring here is an indication that deterioration has occurred in the samples. The respiratory rate increased with the increase in storage duration, and accordingly, decreases in Vitamin C were observed. Javanmardi and Kubota (2006) reported that the change in organic acid content affects the TSS content. A high positive correlation was found between the L* color value and TSS. Higher soluble solid content, which means more sugars, can contribute to a sweeter taste in vegetables. Moreover, some of the pigments responsible for color are water‐soluble, and changes in the concentration of dissolved substances (like sugars) can impact the intensity and perception of color. In other terms, a higher soluble solid content might lead to a sweeter and potentially more vibrant‐colored vegetable. The interplay between sugars and pigments can influence both taste and visual appeal. In this study, it was observed that sugar content increased as Vitamin C content decreased. Significant correlations were observed between vitamin C and the gallic acid, vanillic acid, quercetin, caffeic acid, and chlorogenic acid contents of broccoli samples. Vitamin C can potentially enhance the stability and antioxidant effects of phenolics and flavonoids. Together, they contribute to the overall antioxidant capacity of vegetables.

## 5 CONCLUSION

In conclusion, the postharvest treatment of CA, SA, and PUT could reduce WL and RR in broccoli samples. This research indicated that 0.5 and 1 mM PUT treatments were effective on WL, and 1 mM CA and 1 mM PUT treatments were effective on RR. Moreover, CA, SA, and PUT treatments significantly maintained the postharvest market value of samples by protecting TSS and pH levels and color properties. Compared with control, CA, SA, and PUT treatments gave better results in preserving proximate and mineral composition and biochemical quality characteristics. The study found that treating broccoli samples with 0.5 and 1 mM CA effectively halted the degradation of phenolic and flavonoid compounds. Furthermore, applying 1 mM CA prevented the degradation of vitamin C content. As a result, the use of CA, SA, and PUT as treatments appears promising for preserving postharvest physicochemical contents and extending the storage life of broccoli samples. Therefore, we recommend users to apply 1 mM PUT with 1 mM CA to achieve minimal quality loss while preserving the physicochemical and biochemical compositions of broccoli.

## AUTHOR CONTRIBUTIONS

**Hakan Kibar:** Conceptualization (equal); data curation (equal); methodology (equal); supervision (equal); writing – original draft (equal); writing – review and editing (equal). **Beyhan Kibar:** Data curation (equal); investigation (equal); resources (equal); writing – original draft (equal). **Nezahat Turfan:** Data curation (equal); investigation (equal); methodology (equal); resources (equal).

## FUNDING INFORMATION

This research was not funded.

## CONFLICT OF INTEREST STATEMENT

The authors declare that they do not have any conflicts of interest.

## CONSENT FOR PUBLICATION

All authors have approved the article for publication.

## Data Availability

The data that support the findings of this study are available in the article.
